# Supplementary material for: Impact of COVID-19 social distancing measures on future incidence of invasive pneumococcal disease in England and Wales: a mathematical modelling study
Source: BMJ Open. 2021 Sep 28;11(9):e045380. doi: 10.1136/bmjopen-2020-045380 (PMC8479589; doi:10.1136/bmjopen-2020-045380)
Supplement: Author's manuscript [file bmjopen-2020-045380.draft_revisions.pdf]

BMJ Open

BMJ Open is committed to open peer review. As part of this commitment we make the peer review history of every article we publish publicly available.

When an article is published we post the peer reviewers' comments and the authors' responses online. We also post the versions of the paper that were used during peer review. These are the versions that the peer review comments apply to.

The versions of the paper that follow are the versions that were submitted during the peer review process. They are not the versions of record or the final published versions. They should not be cited or distributed as the published version of this manuscript.

BMJ Open is an open access journal and the full, final, typeset and author-corrected version of record of the manuscript is available on our site with no access controls, subscription charges or pay-per-view fees (http://bmjopen.bmj.com).

If you have any questions on BMJ Open's open peer review process please email info.bmjopen@bmj.com

BMJ Open

Potential impact of Covid-19 response measures on invasive pneumococcal disease in England and Wales

Journal:	*BMJ Open*
Manuscript ID	bmjopen-2020-045380
Article Type:	Original research
Date Submitted by the Author:	29-Sep-2020
Complete List of Authors:	Choi, Yoon Hong; Public Health England, Statistics, Modelling and Economics Department<br>Miller, Elizabeth; London School of Hygiene & Tropical Medicine
Keywords:	COVID-19, Health policy < HEALTH SERVICES ADMINISTRATION & MANAGEMENT, Epidemiology < INFECTIOUS DISEASES, PUBLIC HEALTH

SCHOLARONE™
Manuscripts

I, the Submitting Author has the right to grant and does grant on behalf of all authors of the Work (as defined in the below author licence), an exclusive licence and/or a non-exclusive licence for contributions from authors who are: i) UK Crown employees; ii) where BMJ has agreed a CC-BY licence shall apply, and/or iii) in accordance with the terms applicable for US Federal Government officers or employees acting as part of their official duties; on a worldwide, perpetual, irrevocable, royalty-free basis to BMJ Publishing Group Ltd ("BMJ") its licensees and where the relevant Journal is co-owned by BMJ to the co-owners of the Journal, to publish the Work in this journal and any other BMJ products and to exploit all rights, as set out in our licence.

The Submitting Author accepts and understands that any supply made under these terms is made by BMJ to the Submitting Author unless you are acting as an employee on behalf of your employer or a postgraduate student of an affiliated institution which is paying any applicable article publishing charge ("APC") for Open Access articles. Where the Submitting Author wishes to make the Work available on an Open Access basis (and intends to pay the relevant APC), the terms of reuse of such Open Access shall be governed by a Creative Commons licence – details of these licences and which Creative Commons licence will apply to this Work are set out in our licence referred to above.

Other than as permitted in any relevant BMJ Author's Self Archiving Policies, I confirm this Work has not been accepted for publication elsewhere, is not being considered for publication elsewhere and does not duplicate material already published. I confirm all authors consent to publication of this Work and authorise the granting of this licence.

For peer review only

Potential impact of Covid-19 response measures on invasive pneumococcal disease in England and Wales

Yoon Hong Choi[1] and Elizabeth Miller[2]

1. Statistics and Modelling Economics Department, Public Health England, London, England

2. Department of Infectious Disease Epidemiology, Faculty of Epidemiology & Population Health, London School of Hygiene & Tropical Medicine, London, England

Corresponding author. Yoon.choi@phe.gov.uk

Key words: COVID-19, Social distancing, Pneumococcal transmission model, Invasive pneumococcal disease, Pneumococcal Conjugate Vaccine

For peer review only

Abstract

Objectives

In January 2020, the United Kingdom (UK) moved to a 1+1 schedule for the 13-valent pneumococcal conjugate vaccine (PCV13) with a single priming dose at 3 months and a 12month booster. We modelled the potential impact on invasive pneumococcal disease (IPD) of a drop in PCV13 coverage associated with the restrictions on non-essential health care visits introduced to combat COVID-19 in the UK on 23 March 2020.

Design

Using an existing model of pneumococcal transmission in England and Wales we simulated the impact of a 50% reduction in coverage for 3 months from 23 March without subsequent catch-up vaccination. To implement social distancing, we reduced mixing between and within age-groups by 10% or 50%. In a sensitivity analysis we explored more extreme reductions in coverage.

Main outcome measures

Annual numbers of IPD cases predicted by the model under different vaccination and "lockdown" scenarios with uncertainty intervals (UI) generated from the minimum and maximum values of the model predictions using 500 parameter sets with values within a pre-specified range of the maximum likelihood set.

For peer review only - http://bmjopen.bmj.com/site/about/guidelines.xhtml

Results

The model predicted that any increase in IPD cases from a reduction in PCV13 coverage would be more than offset by a reduction in pneumococcal transmission due to social distancing, with a net reduction in cumulative IPD cases (UI -1,479, -1,061, all ages) over the next five years. Similar results were obtained in the sensitivity analysis, though with a greater reduction with a 6 month "lockdown".

Conclusion

COVID-19 social distancing measures are predicted to have had a profound effect on pneumococcal transmission resulting in a reduction in pneumococcal carriage prevalence and IPD incidence over the first two years after the "lockdown". Carriage studies will be informative in confirming the predicted impact of the social distancing measures after they have been lifted.

Strengths and limitations of this study

For this study we used a previously published realistic dynamic model of pneumococcal transmission that was fitted to past data on age-specific pneumococcal carriage and invasive pneumococcal disease (IPD) and was able to reproduce the observed herd immunity effects of the pneumococcal conjugate vaccination programme in England and Wales.

Our conclusions were robust under the extreme scenario of assuming only a 10% reduction in mixing as a result of the "lockdown" and suspension of all PCV13 vaccination for 3 months with no catch-up.

A limitation of our model is uncertainty over the future behaviour of non-vaccine serotypes although this is unlikely to affect the overall conclusions.

The predicted effect of the reduction in social mixing on carriage of pneumococci could potentially reduce development of natural immunity but we were unable with the current model structure to investigate this.

Introduction

Following the first identification of local transmission of SARS-CoV-2 in England on February 28 [1] the UK government instituted a series of control measures to reduce spread of the virus. On March 18 schools along with restaurants and places of entertainment and leisure were closed, and on March 23 all non-essential travel was banned with members of the population only permitted to

For peer review only - http://bmjopen.bmj.com/site/about/guidelines.xhtml

leave their home for exercise once a day, or in order to obtain food or medication [2]. The public was advised to only use the National Health Service when essential [3] and many general practices replaced face-to-face consultations with an online service [4].

In the UK, paediatric vaccinations are given in general practice and there is concern that the measures taken to limit spread of SARS-CoV-2 may result in many children's vaccinations being postponed with a resulting increase in vaccine-preventable infections [5]. Indeed early coverage data for measles/mumps/rubella vaccine for the first 3 weeks of the "lockdown" in London suggest a drop of over 40% compared with the same period in 2019 [6]. Of particular concern is the impact of a drop in coverage of the 13 valent pneumococcal conjugate vaccine (PCV13) for which the national 2+1 schedule (2, 4, and 12 months) was changed on 1 January 2020 to a 1+1 schedule at 3 and 12 months [7]. The rationale for the schedule change was that after a decade of high coverage with PCV13 much of the protection of young children against vaccine-type invasive pneumococcal disease (IPD) is through herd immunity generated by the 12 month booster dose and that dropping a priming dose would have little effect on this. The supporting evidence for the schedule change came from an immunogenicity study showing similar post-booster antibody responses to PCV13 serotypes with a 2+1 and 1+1 schedule [8] and a modelling study which predicted little impact on IPD cases in children or adults if protection against vaccine-type carriage was similar post-booster with the two schedules [9].

Using our previously described pneumococcal transmission model [9] we investigated the impact on IPD if PCV13 coverage was substantially reduced during the "lockdown" period in the UK. We also took account in the model of the likely impact on pneumococcal transmission of the social mixing restrictions put in place to limit the spread of the SARS-CoV-2 virus.

Model and assumptions.

The same compartmental deterministic model and estimated parameter values as described by Choi et al. [9] were used in this study. Briefly, the model simulates the transmission dynamics of carried pneumococci between and within different age groups and predicts carriage changes by serotype grouping, either vaccine type (VT) or non-vaccine type (NVT), under different vaccination scenarios. Carriage changes are then translated into IPD cases using case-carrier ratios (CCRs) that describe the invasive potential of different carried serotypes. The model has 26 parameters which describe the propensity for vaccine-induced replacement of VTs with NVTs in carriage, and the CCRs, in different age groups. The maximum likelihood values of these parameters and their uncertainty ranges are estimated by fitting the model to IPD data up to the end of the epidemiological year from July 2015

For peer review only - http://bmjopen.bmj.com/site/about/guidelines.xhtml

to June 2016 [9]. In implementing the effect of the 1+1 schedule, the model described by Choi et al. [9] assumed that one priming dose at 3 months of age provides half the protection against VT carriage as two doses or, under a more conservative assumption, that a single priming dose provides no protection against VT carriage. The latter assumption was not included in the current analysis; in addition the change to the 1+1 PCV13 schedule reflected the actual start date of January 2020, not the assumed date of September 2018 as in the original model.

We assumed as a base case a 3 month “lockdown” period starting from March 23 during which there was a 50% reduction in coverage for children who would otherwise have received a priming or booster dose of PCV13 during this period and that there would be no subsequent catch up vaccination for these children. To implement the impact of reduced social mixing we used the same mixing matrix as before [9] based on the POLYMOD study [10] but reduced the number of contacts between and within each age group by either 10% or 50%. In a sensitivity analysis we explored an extreme scenario of cessation of all PCV13 vaccination for the duration of the 3 month “lockdown”, and of extending the duration of the “lockdown” for 6 months with 50% reduction in coverage.

Results are shown as the median of the model outputs under the different scenarios. The uncertainty interval (UI) for each scenario represents the minimum and maximum values generated with 500 parameters sets in which each of the 26 model parameters had values within a pre-specified range (±0.3) of the maximum likelihood set [9]. The impact on IPD of the temporary changes in PCV13 coverage and social mixing is presented by VT, which includes PCV13 serotypes minus serotypes 1 and 3, and NVT; serotype 3 is included with the NVT group in the model on the grounds that it has shown an increase post-PCV13 similar to that seem for NVTs [9].

Patient and Public Involvement statement: Our study did not involve any human participation.

Results

Figure 1 shows the predicted number of IPD cases in England and Wales by epidemiological year out to 2030/31 in under 5 year olds and in 65+ year olds by VT and NVT group assuming a 50% reduction in PCV13 coverage and a 10% or 50% reduction in contact rates for 3 months. Even with only a 10% reduction in contact rates, the predicted impact of reduced mixing outweighed any increase in IPD due to reduction in PCV13 coverage. The longer term increases in NVT IPD cases in those aged 65 years and over is the consequence of the increasing mean age of this population and the steep increase in IPD incidence with age in this group. Under the extreme assumption that none of the children eligible for PCV13 during the 3 month “lockdown” was ever vaccinated, the results still showed an overall reduction in IPD cases in under 5 year olds and 65+ year olds (Fig S1, Appendix).

For peer review only - http://bmjopen.bmj.com/site/about/guidelines.xhtml

The predicted beneficial effect of the lockdown on IPD cases was even more marked with a 6 month “lockdown” period (Fig S2, Supplementary Appendix).

The cumulative number of additional IPD cases predicted in England and Wales over the first five epidemiological years starting from July 2019 is shown in Table 1 by age group and serotype grouping for a 3 month” lockdown” period and a 10% reduction in contact rates. Across all ages the model predicted a net saving of 1,239 IPD cases (UI -1,479, -1,061) over this period compared with the predicted number of IPD cases without the “lockdown”. Without any reduction in contact rates, under the worst case of no PCV13 vaccination for 6 months and no catch up, the model predicted an additional 274 (UI 136, 450) VT IPD cases, of which 27 (UI 14, 44) were in children under 2 years of age. The overall predicted increase in IPD cases was smaller (208 (UI 102, 338)) due to a concomitant reduction in NVT IPD cases.

Discussion

In line with many other countries, the UK adopted a “lockdown” strategy to minimise social contacts and thereby reduce the spread of the SARS-CoV-2 virus. The restriction on non-essential travel and GP visits raised concerns that vaccine coverage in children might be substantially reduced [5], echoing similar concerns raised at the global level [11]. With the recent change to a 1+1 PCV13 schedule in the UK, which is reliant on the booster dose to maintain herd immunity [8,9], a substantial reduction in PCV13 coverage could compromise control of IPD. Our modelling study suggests that any potential impact of the social distancing policy on PCV13 coverage and herd immunity will be more than offset by a reduction in pneumococcal transmission such that a net reduction in IPD cases should occur. This reduction in IPD would be the result of an overall reduction in pneumococcal carriage prevalence over the first two years after the lockdown, unlike the IPD reduction achieved with PCV. Carriage studies in England before and after the introduction of PCVs have shown little impact on overall carriage prevalence with the reduction in carriage of vaccine serotypes offset by an increase in non-vaccine serotypes [12]. The overall reduction in IPD cases achieved by PCVs is the consequence of the generally lower CCR of the NVTs that replaced VTs in carriage.

Invasive pneumococcal disease is a consequence, albeit rare, of carriage of *Streptococcus pneumoniae* in the nasopharynx. Acquisition of the pneumococcus is by person-to-person transmission and occurs via respiratory droplets with transmission enhanced in settings with close mixing such as day care centres and military camps [13]. Visits to the GP for mild upper respiratory tract disease have also been shown to be a risk factor for pneumococcal acquisition [14]. It is

For peer review only - http://bmjopen.bmj.com/site/about/guidelines.xhtml

therefore to be expected that the measures taken to reduce spread of SARS-CoV-2 would also reduce pneumococcal transmission. A study of the social contacts of 1,356 UK adults conducted between 24 and 27 March suggested a 74% reduction [15] compared with the historical rates documented in the POLYMOD study [10]. However, this study could not assess the impact on contacts made by children, particularly those of pre-school age who are the main drivers of pneumococcal transmission [9]. Hence, we adopted a more conservative range for the reduction in social contacts across all ages in our model. Nevertheless, even with only a 10% reduction in mixing a substantial net reduction in IPD cases over the next 5 years was predicted.

Whether a reduction in coverage caused by the “lockdown” would have a similar effect on other vaccine-preventable infections cannot be assessed with our model as it is specific to pneumococcal infection. However, the average number of weekly notifications of whooping cough and meningococcal septicaemia in England in the 18 weeks from 23 March 2020 showed a 80% and 59% reduction respectively compared with the weekly average for the same 18 week period in 2019 [16]. As with IPD, nasopharyngeal colonisation is a pre-requisite for whooping cough and meningococcal disease. While the completeness of notifications by doctors may have suffered somewhat as a result of the COVID-19 epidemic, the magnitude of the decline in these two other infections is consistent with a substantial reduction in new colonisation episodes following the “lockdown” and suggests that, at least in the short term, this has outweighed any potential increase due to a reduction in coverage of pertussis or meningococcal vaccines. For measles, which is highly infectious, small reductions in coverage can have more of an impact as shown by the loss of the UK’s former measles elimination status in 2019 following relatively small declines in vaccine coverage that allowed the re-establishment of endemic measles transmission [17]. While measles notifications have shown an 84% decline since the start of the “lockdown” many such notifications turn out not measles when laboratory confirmation is sought [16]. As with IPD, published data on confirmed measles cases during the “lockdown” are not yet available and may be difficult to interpret due to interruption of the normal processes that lead to laboratory confirmation of suspected cases during the COVID-19 epidemic.

Our model has limitations as previously described [9]. These include uncertainty over the future behaviour of non-vaccine serotypes and in the mixing patterns among the oldest age groups. However, given the magnitude of the predicted effect of the “lockdown”, these uncertainties are unlikely to materially affect the results. It is also possible that the reduction in pneumococcal carriage predicted to occur as a result of the “lockdown” may affect induction of natural immunity. Nasopharyngeal carriage can boost serotype-specific IgG antibody levels to the carried serotype and

For peer review only - http://bmjopen.bmj.com/site/about/guidelines.xhtml

such naturally induced capsular antibodies have been shown to protect against carriage of that serotype [18]. If natural immunity against carriage was reduced as a result of decreased pneumococcal transmission this could offset the reduction in IPD predicted to persist after relaxation of the “lockdown”. Our model does not include a natural immunity compartment, so we were unable to explore the potential effect of such perturbations in carriage-induced immunity.

In conclusion, our pneumococcal model predicts that the effect of the COVID-19 “lockdown” on pneumococcal carriage rates will result in a reduction in IPD cases over the next two years, thus obscuring the predicted small increase in VT IPD due to the adoption of a 1+1 PCV13 schedule [9]. Pneumococcal carriage studies conducted over the next few years could help elucidate the impact of the social distancing measures and of the adoption of the 1+1 PCV13 schedule on the future incidence of VT and NVT IPD.

References

1 Coronavirus: First Covid-19 transmission within the UK confirmed | inews. https://inews.co.uk/news/uk/coronavirus-first-covid-19-transmission-uk-confirmed-2004500 (accessed 31 Jul 2020).

2 COVID-19: guidance on social distancing and for vulnerable people - GOV.UK. https://www.gov.uk/government/publications/covid-19-guidance-on-social-distancing-and-for-vulnerable-people (accessed 31 Jul 2020).

3 NHS. Staying at home and away from others (social distancing) - GOV.UK. https://www.gov.uk/government/publications/full-guidance-on-staying-at-home-and-away-from-others/full-guidance-on-staying-at-home-and-away-from-others (accessed 31 Jul 2020).

4 How to get medical help from home - Coronavirus (COVID-19) - NHS. https://www.nhs.uk/conditions/coronavirus-covid-19/staying-at-home-to-avoid-getting-coronavirus/how-to-get-medical-help-from-home/ (accessed 31 Jul 2020).

5 Missed vaccinations could lead to other fatal outbreaks, doctors warn | Society | The Guardian. https://www.theguardian.com/society/2020/apr/26/missed-vaccinations-could-lead-to-other-fatal-outbreaks-doctors-warn (accessed 31 Jul 2020).

For peer review only

6 McDonald HI, Tessier E, White JM, et al. Early impact of the coronavirus disease (COVID-19) pandemic and physical distancing measures on routine childhood vaccinations in England, January to April 2020. Eurosurveillance 2020;25:2000848. doi:10.2807/1560-7917.ES.2020.25.19.2000848

7 Changes to the infant pneumococcal conjugate vaccine schedule. https://assets.publishing.service.gov.uk/government/uploads/system/uploads/attachment_data/file/854153/Letter_NHSEI_PHE_PCV13_schedule_change.pdf (accessed 31 Jul 2020).

8 Goldblatt D, Southern J, Andrews NJ, et al. Pneumococcal conjugate vaccine 13 delivered as one primary and one booster dose (1 + 1) compared with two primary doses and a booster (2 + 1) in UK infants: a multicentre, parallel group randomised controlled trial. Lancet Infect Dis 2018;18:171–9. doi:10.1016/S1473-3099(17)30654-0

9 Choi YH, Andrews N, Miller E. Estimated impact of revising the 13-valent pneumococcal conjugate vaccine schedule from 2+1 to 1+1 in England and Wales: A modelling study. PLOS Med 2019;16:e1002845. doi:10.1371/journal.pmed.1002845

10 Mossong JJ, Hens N, Jit M, et al. Social contacts and mixing patterns relevant to the spread of infectious diseases. PLoS Med 2008;5:e74.

11 Hirabayashi K. The impact of COVID-19 on routine vaccinations: Reflections during World Immunization Week 2020. https://www.unicef.org/eap/stories/impact-covid-19-routine-vaccinations (accessed 31 Jul 2020).

12 Southern J, Andrews N, Sandu P, et al. Pneumococcal carriage in children and their household contacts six years after introduction of the 13- valent pneumococcal conjugate vaccine in England. PLoS One 2018;13:e0195799.

13 Sleeman KL, Daniels L, Gardiner M, et al. Acquisition of Streptococcus pneumoniae and Nonspecific Morbidity in Infants and Their Families. Pediatr Infect Dis J 2005;24:121–7. doi:10.1097/01.inf.0000151030.10159.b1

14 Jarvis CI, Van Zandvoort K, Gimma A, et al. Quantifying the impact of physical distance measures on the transmission of COVID-19 in the UK. BMC Med 2020;18:124. doi:10.1186/s12916-020-01597-8

15 Jarvis CI, Van Zandvoort K, Gimma A, et al. Quantifying the impact of physical distance measures on the transmission of COVID-19 in the UK. BMC Med 2020;18:124. doi:10.1186/s12916-020-01597-8

For peer review only - http://bmjopen.bmj.com/site/about/guidelines.xhtml

16 Public Health England. Notifiable diseases: last 52 weeks - GOV.UK. https://www.gov.uk/government/publications/notifiable-diseases-last-52-weeks (accessed 31 Jul 2020).

17 Public Health England. Measles in England - Public health matters. https://publichealthmatters.blog.gov.uk/2019/08/19/measles-in-england/ (accessed 31 Jul 2020).

18 Goldblatt D, Hussain M, Andrews N, et al. Antibody responses to nasopharyngeal carriage of Streptococcus pneumoniae in adults: A longitudinal household study. J Infect Dis 2005;192:387–93. doi:10.1086/431524

For peer review only

Table 1. Cumulative difference in cases of invasive pneumococcal disease over 5 epidemiological years (July to June) starting from 2019/2020 by age group and serotype grouping with a 50% reduction in PCV13 coverage, 10% reduction in contact rates and 3 month “lockdown” period starting 23 March 2020. Results show median and minimum and maximum of the range of model outputs generated by 500 best fitting parameter sets [9]

	0-1Y	2-4Y	5-14Y	15-44Y	45-65Y	65Y+	Overall
VT	0<br>(-1,1)	0<br>(0,0)	-2<br>(-3,-1)	-18<br>(-31,-8)	-22<br>(-39,-11)	-38<br>(-66,-18)	-80<br>(-140,-36)
NVT	-36<br>(-46,-26)	-13<br>(-16,-9)	-23<br>(-30,-21)	-172<br>(-195,-157)	-295<br>(-365,-252)	-621<br>(-735,-522)	-1,160<br>(-1,388,-987)
Overall	-36<br>(-46,-26)	-13<br>(-17,-9)	-25<br>(-32,-23)	-190<br>(-216,-174)	-317<br>(-390,-273)	-658<br>(-776,-557)	-1,239<br>(-1,476,-1,061)

For peer review only

For peer review only - http://bmjopen.bmj.com/site/about/guidelines.xhtml

Figure 1. Annual number of IPD cases by VT and NVT and epidemiological year (July to June) from 2018/19 to 2030/31 with a 50% reduction in PCV13 coverage for 3 months starting 23 March 2020 obtained from the long-term simulation model: A. Under 5 year olds with 10% reduction in contact rates: B. Under 5 year olds with 50 % reduction in contact rates. C. 65+ year olds with 10% reduction in contact rates, D. 65+ year olds with 50% reduction in contact rates (Width of lines indicates uncertainty intervals). (IPD, Invasive pneumococcal disease; VT, Vaccine types; NVT, Non-vaccine types)

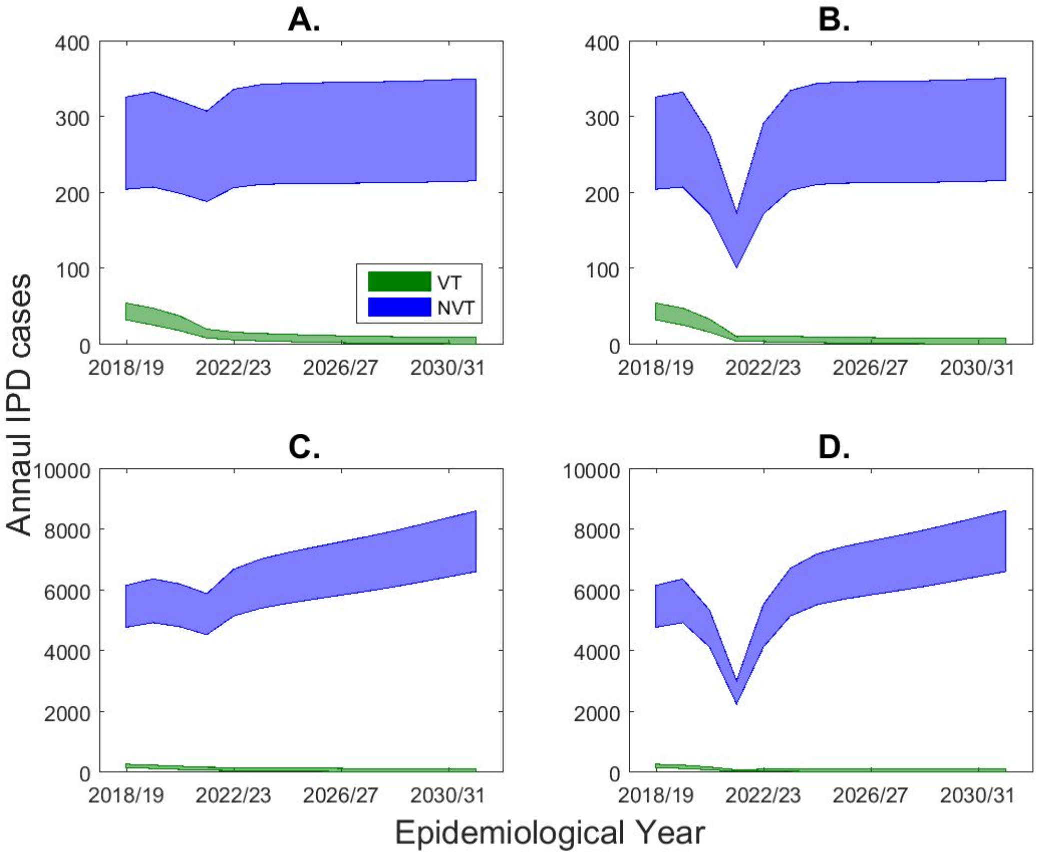

For peer review only - http://bmjopen.bmj.com/site/about/guidelines.xhtml

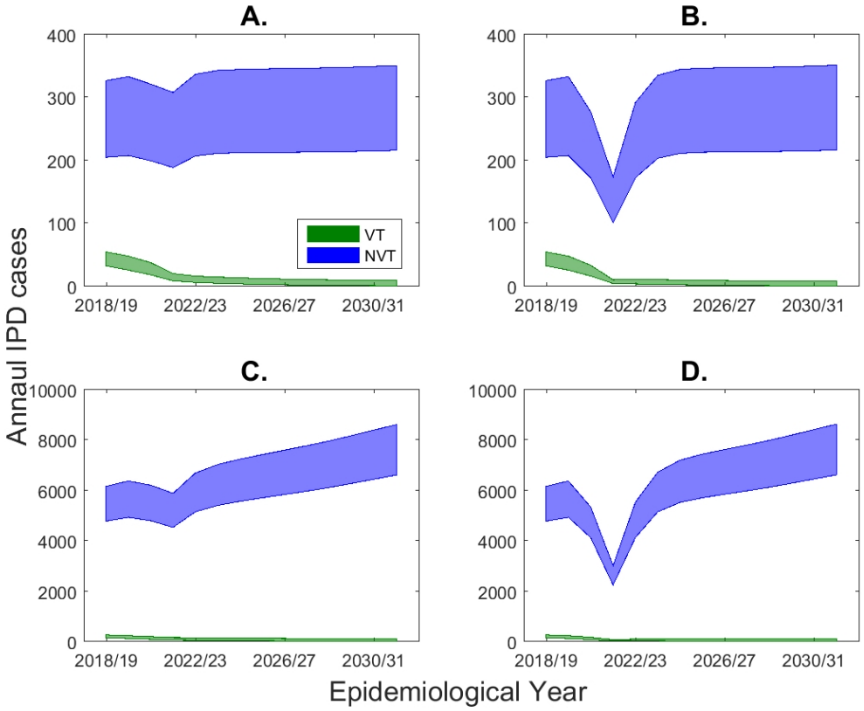

Figure 1. Annual number of IPD cases by VT and NVT and epidemiological year (July to June) from 2018/19 to 2030/31 with a 50% reduction in PCV13 coverage for 3 months starting 23 March 2020 obtained from the long-term simulation model: A. Under 5 year olds with 10% reduction in contact rates: B. Under 5 year olds with 50 % reduction in contact rates. C. 65+ year olds with 10% reduction in contact rates, D. 65+ year olds with 50% reduction in contact rates (Width of lines indicates uncertainty intervals). (IPD, Invasive pneumococcal disease; VT, Vaccine types; NVT, Non-vaccine types)

203x152mm (150 x 150 DPI)

Supplementary Appendix

Figure S1. Annual number of IPD cases by VT and NVT and epidemiological year (July to June) from 2018/19 to 2030/31 with a 100% reduction in PCV13 coverage for 3 months starting 23 March 2020 obtained from the long-term simulation model; A. Under 5 year olds with 10% reduction in contact rates: B. Under 5 year olds with 50 % reduction in contact rates. C. 65+ year olds with 10% reduction in contact rates, D. 65+ year olds with 50% reduction in contact rates. rates (Width of lines indicates uncertainty intervals). (IPD, Invasive pneumococcal disease; VT, Vaccine types; NVT, Non-vaccine types)

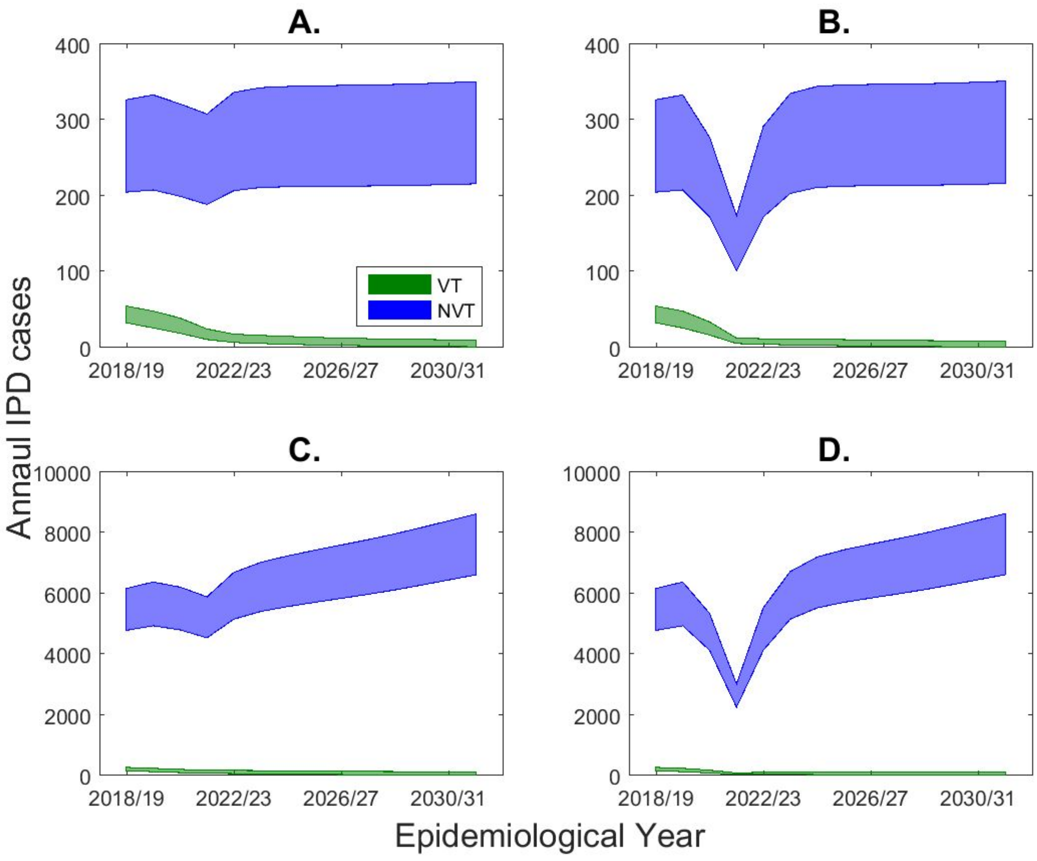

Figure S2. Annual number of IPD cases by VT and NVT and epidemiological year (July to June) from 2018/19 to 2030/31 with a 50 % reduction in PCV13 coverage for 6 months starting 23 March 2020 obtained from the long-term simulation model; A. Under 5 year olds with 10% reduction in contact rates: B. Under 5 year olds with 50 % reduction in contact rates. C. 65+ year olds with 10% reduction in contact rates, D. 65+ year olds with 50% reduction in contact rates (Width of lines indicates uncertainty intervals). (IPD, Invasive pneumococcal disease; VT, Vaccine types; NVT, Non-vaccine types)

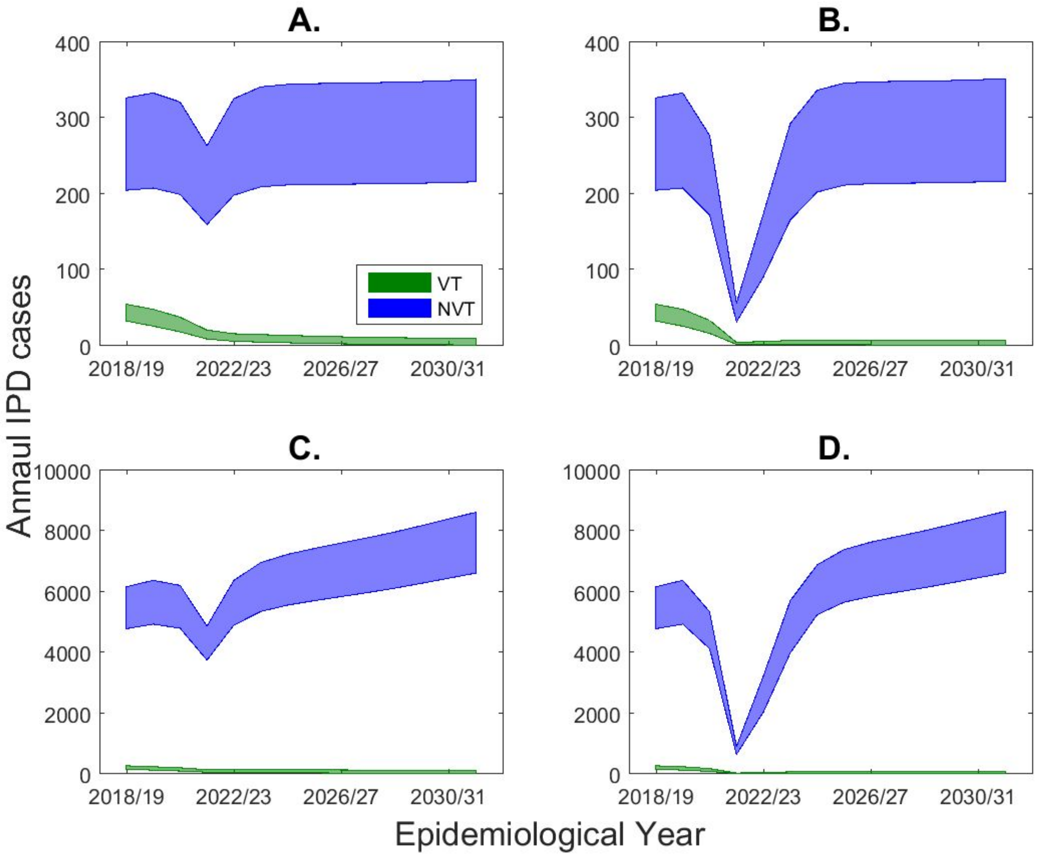

BMJ Open

Impact of Covid-19 social distancing measures on future incidence of invasive pneumococcal disease in England and Wales – a mathematical modelling study

Journal:	*BMJ Open*
Manuscript ID	bmjopen-2020-045380.R1
Article Type:	Original research
Date Submitted by the Author:	10-May-2021
Complete List of Authors:	Choi, Yoon Hong; Public Health England, Statistics, Modelling and Economics Department<br>Miller, Elizabeth; London School of Hygiene & Tropical Medicine
**Primary Subject Heading**:	Public health
Secondary Subject Heading:	Epidemiology, Infectious diseases, Qualitative research
Keywords:	COVID-19, PUBLIC HEALTH, INFECTIOUS DISEASES, EPIDEMIOLOGY

SCHOLARONE™
Manuscripts

I, the Submitting Author has the right to grant and does grant on behalf of all authors of the Work (as defined in the below author licence), an exclusive licence and/or a non-exclusive licence for contributions from authors who are: i) UK Crown employees; ii) where BMJ has agreed a CC-BY licence shall apply, and/or iii) in accordance with the terms applicable for US Federal Government officers or employees acting as part of their official duties; on a worldwide, perpetual, irrevocable, royalty-free basis to BMJ Publishing Group Ltd ("BMJ") its licensees and where the relevant Journal is co-owned by BMJ to the co-owners of the Journal, to publish the Work in this journal and any other BMJ products and to exploit all rights, as set out in our licence.

The Submitting Author accepts and understands that any supply made under these terms is made by BMJ to the Submitting Author unless you are acting as an employee on behalf of your employer or a postgraduate student of an affiliated institution which is paying any applicable article publishing charge ("APC") for Open Access articles. Where the Submitting Author wishes to make the Work available on an Open Access basis (and intends to pay the relevant APC), the terms of reuse of such Open Access shall be governed by a Creative Commons licence – details of these licences and which Creative Commons licence will apply to this Work are set out in our licence referred to above.

Other than as permitted in any relevant BMJ Author's Self Archiving Policies, I confirm this Work has not been accepted for publication elsewhere, is not being considered for publication elsewhere and does not duplicate material already published. I confirm all authors consent to publication of this Work and authorise the granting of this licence.

For peer review only

Impact of Covid-19 social distancing measures on future incidence of invasive pneumococcal disease in England and Wales – a mathematical modelling study

Yoon Hong Choi[1*] and Elizabeth Miller[2]

1. Statistics and Modelling Economics Department, Public Health England, London, England

2. Department of Infectious Disease Epidemiology, Faculty of Epidemiology & Population Health, London School of Hygiene & Tropical Medicine, London, England

* Corresponding author

Address: Modelling and Economics Unit, Statistics and Modelling Economics Department, Public Health England, 61 Colindale Ave, London, NW9 5EQ, United Kingdom

E-mail: Yoon.choi@phe.gov.uk

Tel: +44 (0) 20 8327 7454

Fax: +44 (0) 20 8200 4400

For peer review only

Key words: COVID-19, Social distancing, Lockdown, Pneumococcal transmission model, Invasive pneumococcal disease, Pneumococcal Conjugate Vaccine

Abstract

Objectives

In January 2020, the United Kingdom moved to a 1+1 schedule for the 13-valent pneumococcal conjugate vaccine (PCV13) with a single priming dose at 3 months and a 12month booster. We modelled the impact on invasive pneumococcal disease (IPD) out to 2030/31 of reductions in PCV13 coverage and population mixing associated with restrictions on non-essential health care visits and social distancing measures introduced in 2020/21 to reduce SARS-CoV-2 transmission.

Design

Using an existing model of pneumococcal transmission in England and Wales we simulated the impact of a 40% reduction in coverage and a 40% reduction in mixing between and within age-

groups during two lockdowns in spring 2020 and autumn/winter 2020/21. More and less extreme reductions in coverage and mixing were explored in a sensitivity analysis.

Main outcome measures

Predicted annual numbers of IPD cases under different coverage and mixing reduction scenarios with uncertainty intervals (UI) generated from minimum and maximum values of the model predictions using 500 parameter sets.

Results

The model predicted that any increase in IPD cases resulting from a reduction in PCV13 coverage would be more than offset by a reduction in pneumococcal transmission due to social distancing measures and that overall reductions in IPD cases will persist for a few years after resumption of normal mixing. The net reduction in cumulative IPD cases over the five epidemiological years from July 2019 was predicted to be 13,494 (UI 12,211, 14,676) all ages. Similar results were obtained in the sensitivity analysis.

Conclusion

COVID-19 lockdowns are predicted to have had a profound effect on pneumococcal transmission resulting in a reduction in pneumococcal carriage prevalence and IPD incidence for up to five years after the end of the lockdown period. Carriage studies will be informative in confirming the predicted impact of the lockdown measures after they have been lifted.

Strengths and limitations of this study

For this study we used a previously published dynamic model of pneumococcal transmission that was fitted to past data on age-specific pneumococcal carriage and IPD and was able to reproduce the observed herd immunity effects of the pneumococcal conjugate vaccination programme in England and Wales. Short term model predictions of the effect of the first lockdown were validated against IPD data in England and Wales to June 2020.

Our conclusions were robust under the extreme scenario of assuming only a 10% reduction in mixing as a result of the two lockdowns and suspension of all PCV13 vaccination during the lockdown periods with no subsequent catch-up vaccination.

A limitation of our model is uncertainty over the future behaviour of non-vaccine serotypes although this is unlikely to affect the overall conclusions.

For peer review only - http://bmjopen.bmj.com/site/about/guidelines.xhtml

The predicted effect of the reduction in social mixing on carriage of pneumococci could potentially reduce development of natural immunity as a result of carriage but we were unable with the current model structure to investigate this.

For peer review only

For peer review only - http://bmjopen.bmj.com/site/about/guidelines.xhtml

Introduction

Following the first identification of local transmission of SARS-CoV-2 in England on February 28, 2020, [1] the United Kingdom (UK) government instituted a series of control measures to reduce spread of the virus. On March 18 schools along with restaurants and places of entertainment and leisure were closed, and on March 23 all non-essential travel was banned with members of the population only permitted to leave their home for exercise once a day, or in order to obtain food or medication [2]. The public was advised to only use the National Health Service when essential [3] and many general practices replaced face-to-face consultations with an online service [4]. This first national lockdown was eased in stages starting in May 2020 with children returning to schools in September 2020 [5]. As COVID-19 cases started to increase again in September 2020, a variety of locally-based measures were introduced in areas of increasing incidence in an attempt to reduce transmission. Despite these local measures cases continued to increase and a second national lockdown came into force in November 2020 which lasted for six months until May 2021 [5].

In the UK, paediatric vaccinations are given in general practice and there was initial concern that the measures taken to limit spread of SARS-CoV-2 would result in many children's vaccinations being postponed with a resulting increase in vaccine-preventable infections [6]. Indeed early coverage data for measles/mumps/rubella vaccine for the first 3 weeks of the first lockdown in London suggested a drop of over 40% compared with the same period in 2019 [7]. Of particular concern is the impact of a drop in coverage of the 13 valent pneumococcal conjugate vaccine (PCV13) for which the national 2+1 schedule (2, 4, and 12 months) was changed on 1 January 2020 to a 1+1 schedule at 3 and 12 months [8]. The rationale for the schedule change was that after a decade of high coverage with PCV13 much of the protection of young children against vaccine-type invasive pneumococcal disease (IPD) is through herd immunity generated by the 12 month booster dose and that dropping a priming dose would have little effect on this. The supporting evidence for the schedule change came from an immunogenicity study showing similar post-booster antibody responses to PCV13 serotypes with a 2+1 and 1+1 schedule [9] and a modelling study which predicted little impact on IPD cases in children or adults if protection against vaccine-type carriage was similar post-booster with the two schedules [10].

Using our previously described pneumococcal transmission model [10] we investigated the impact on IPD if PCV13 coverage was substantially reduced during the lockdown periods in the UK. The model also allowed us to investigate the likely longer-term impact on pneumococcal transmission of social mixing restrictions put in place to limit the spread of the SARS-CoV-2 virus in England and Wales.

For peer review only - http://bmjopen.bmj.com/site/about/guidelines.xhtml

Model and assumptions.

The same compartmental deterministic model and estimated parameter values as described by Choi et al. [10] were used in this study. Briefly, the model simulates the transmission dynamics of carried pneumococci between and within different age groups and predicts carriage changes by serotype grouping, either vaccine type (VT) or non-vaccine type (NVT), under different vaccination scenarios. Carriage changes are then translated into IPD cases using case-carrier ratios (CCRs) that describe the invasive potential of different carried serotypes. The model has a Susceptible-Infected-Susceptible (SIS) structure as it is assumed that a carriage episode does not generate any serogroup-specific protection. The model has 26 parameters which describe the propensity for vaccine-induced replacement of VTs with NVTs in carriage, the CCRs in different age groups, and the effectiveness of PCV vaccination against carriage of different vaccine serotypes. (see Supplementary Appendix for summary of model description and parameter estimation). The maximum likelihood values of these parameters and their uncertainty intervals are estimated by fitting the model to IPD data up to the end of the epidemiological year from July 2015 to June 2016 [10]. In implementing the effect of the 1+1 schedule, the base case in the model described by Choi et al. [10] assumed that one priming dose at 3 months of age provides half the protection against VT carriage as two doses while the effect of the 12 month booster remains unchanged. In the current analysis, the change to the 1+1 PCV13 schedule was implemented to reflect the actual start date of January 2020, not the assumed date of September 2018 as in the original model [10].

We assumed that, during the first two-month lockdown period starting from 23rd March 2020 and also during the second six-month lockdown starting from 5th November 2020, there was a 40% reduction in PCV13 coverage, as suggested by early declines in MMR vaccine coverage in London during the first lockdown [7]. For the sensitivity analysis we assumed two additional scenarios, one with no material decline in PCV13 coverage (0% decline) and a second with cessation of all PCV13 vaccination during the lockdown periods (100% decline). The 0% scenario was chosen to reflect a situation in which there was a rapid catch-up of missed PCV13 vaccinations after the first lockdown with no reduction during the second while the 100% reduction scenario was explored as an extreme scenario. We pessimistically assumed there would be no subsequent catch up vaccination for children under the 40% and 100% reduction scenarios as final coverage data for the affected birth cohorts will not be available until 2021/22.

To implement the impact of reduced social mixing during each of the lockdown periods we used the same mixing matrix as before [10] based on the POLYMOD study [11] and an additional contact survey among infants under one year [12] but reduced the number of contacts between and within

For peer review only - http://bmjopen.bmj.com/site/about/guidelines.xhtml

each age group by 40% as a baseline scenario and explored 10% and 74% reduction scenarios for the sensitivity analysis. The 74% reduction scenario was based on early data from a study of the social contacts of 1,356 UK adults between 24 and 27 March [13] compared with the historical rates documented in the POLYMOD study [11]. However, this study [13] could not assess the impact on contacts made by children, particularly those of pre-school age who are the main drivers of pneumococcal transmission [10]. As the social distancing measures in children were less strict than adults, the estimated reduction in mixing would be lower in children and so we took the 74% scenario as the upper extreme scenario for the contact pattern reduction.

Results are shown as the median of the model outputs under the different scenarios. The uncertainty interval (UI) for each scenario represents the minimum and maximum values generated with 500 model parameters sets obtained from Choi et al. [10]. The impact on IPD of the temporary changes in PCV13 coverage and social mixing is presented by VT, which includes PCV13 serotypes minus serotypes 1 and 3, and NVT; serotype 3 is included with the NVT group in the model on the grounds that it has shown an increase post-PCV13 similar to that seem for NVTs while serotype 1 is excluded from the model due to secular changes which are independent of vaccine usage [10] .

Patient and Public Involvement statement: Our study did not involve any direct participation by human subjects.

Results

Fig 1 shows the predicted number of IPD cases in England and Wales by epidemiological year out to 2030/31 in under 5 year olds and in 65+ year olds by VT and NVT group with four different scenarios. The first scenario presented in Figs 1 A&B is without any change in PCV13 coverage and contact patterns; the increase in IPD cases out to 2030/31 in 65+ year olds reflects the increasing mean age of individuals in this age group in the future together with the steep increase in IPD incidence with increasing age in the elderly population. The remaining three scenarios in Figs 1 C-H assume a 40% reduction in PCV13 coverage with respectively a 10% (C&D), 40% (E&F) or 74% (G&H) reduction in contact rates during the two lockdowns. Even with only a 10% reduction in contact rates in Figs 1 C&D, the predicted impact of reduced social mixing outweighed any increase in IPD due to reduction in PCV13 coverage. The predicted beneficial effect of the lockdown on IPD cases was even more marked with higher reductions in social mixing (Figs 1 E, F, G and H). Under all the scenarios, the model predicted that the impact of the two lockdowns on IPD would persist for up to five years after their cessation.

For peer review only

For peer review only - http://bmjopen.bmj.com/site/about/guidelines.xhtml

Table 1. Cumulative difference in cases of invasive pneumococcal disease over 5 epidemiological years (July to June) starting from 2019/2020 by age group and serotype grouping (VT, NVT and overall) with a 40% reduction in PCV13 coverage, 40% reduction in contact rates during the two lockdown periods starting from 23 March for two months and from 5 November 2020 for six months. Results show median and minimum and maximum of the range of model outputs generated by 500 model parameter sets [10]. (VT, Vaccine types; NVT, Non-vaccine types)

	0-1Y	2-4Y	5-14Y	15-44Y	45-65Y	65Y+	Overall
VT	-13 (-26, -6)	-2 (-4, -1)	-8 (-15, -3)	-82 (-164, -36)	-94 (-186, -41)	-156 (-312, -69)	-354 (-707, -156)
NVT	-456 (-564, -334)	-167 (-206, -116)	-268 (-343, -249)	-1977 (-2195, -1836)	-3282 (-3976, -2844)	-6938 (-7956, -6053)	-13123 (-14275, -11893)
Overall	-471 (-578, -347)	-169 (-208, -118)	-275 (-349, -256)	-2061 (-2291, -1913)	-3367 (-4062, -2931)	-7107 (-8135, -6201)	-13494 (-14676, -12211)

The cumulative number of additional IPD cases predicted in England and Wales over the first five epidemiological years starting from July 2019 is shown in Table 1 by age group and serotype grouping for a 40% reduction in contact rates and 40% reduction in PCV13 coverage during two lockdowns (base case scenario). Across all ages the model predicted a net saving of 13,494 IPD cases (UI 12,211, 14,676) over this period compared with the predicted number of IPD cases without any lockdowns. The results over the first five years by age group and different scenarios for the reduction in coverage and social mixing are presented in Table S1. Without any reduction in contact rates under the worst case of no PCV13 vaccination during the two lockdown periods and no catch up vaccination, the model predicted a cumulative additional 105 (UI 49, 197) VT IPD cases, of which 14 (UI 6, 26) were in children under 2 years of age over the first five epidemiological years since 2019/20. The overall predicted increase in IPD cases was smaller (73 (UI 33, 130)) due to a concomitant reduction in replacement with NVT IPD cases (Fig 2). While the predicted overall increase is small the perturbation in VT IPD cases as a result of the temporary interruption of vaccination is predicted to persist out to 2030/31.

Amin-Chowdbury et al. [14] reported that there were 2,440 IPD cases in England and Wales between February and June 2019 and 1,137 IPD cases during the same period in 2020 showing a 53%

For peer review only - http://bmjopen.bmj.com/site/about/guidelines.xhtml

reduction in overall IPD cases in England and Wales. The model predicted that there would be 2,350 (UI 2,129, 2,531) IPD cases in the same period in 2019.

Table 2 shows the model predictions for the reduction in IPD cases in February to June 2020 with a 40% reduction in PCV13 coverage during the two lockdown periods and under various reduced mixing scenarios. The scenario with a 40% reduction in mixing pattern most closely matched the observed reduction in IPD during February to June 2020 [14]. Table S2 shows the predicted number of IPD cases during February to June 2020 with different assumptions about reductions in PCV13 coverage and in social mixing. Only the scenarios with a 40% reduction in mixing produced the observed reductions seen in IPD incidence with little impact of varying the PCV13 coverage reductions.

Table 2. Model outputs for the number of overall IPD cases between February and June 2020 and proportional reduction compared to the model predictions for the same period in 2019 (2,350 (UI 2,129, 2,531) IPD cases) in England and Wales according to different reduction levels in contact patterns with 40% PCV13 coverage reduction rate during the first two lockdown starting 23 March for 2 months. s in the UK. Results show median and minimum and maximum of the range of model outputs generated by 500 model parameter sets [10]. (IPD, Invasive pneumococcal disease)

Reduction in mixing	2020	Proportional reduction
0%	2,374(2,146, 2,554)	-0.9%(-1.3%, -0.6%)
10%	1,954(1,766, 2,102)	16.9%(16.5%, 17.3%)
40%	1,125(1,017, 1,211)	52.1%(51.8%, 52.4%)
74%	677(612, 728)	71.2%(71.1%, 71.3%)

Discussion

In line with many other countries, the UK adopted a lockdown strategy to minimise social contacts and thereby reduce the spread of the SARS-CoV-2. The restriction on non-essential travel and GP visits raised concerns that vaccine coverage in children might be substantially reduced [6], echoing similar concerns raised at the global level [15]. With the recent change to a 1+1 PCV13 schedule in the UK, which is reliant on the booster dose to maintain herd immunity [9,10], a substantial reduction in PCV13 coverage could compromise control of IPD. Our modelling study suggests that any potential impact of the lockdown measures on PCV13 coverage and herd immunity will be more than offset by a reduction in pneumococcal transmission due to the reduction in population mixing such that a net reduction in IPD cases should occur. Our model also predicts that the net reduction in

For peer review only - http://bmjopen.bmj.com/site/about/guidelines.xhtml

For peer review only

IPD cases will be sustained for up to five years after mixing has returned to normal levels in the population. This reduction in IPD would be the result of an overall reduction in pneumococcal carriage prevalence after the two lockdown periods, unlike the IPD reduction achieved with PCV. Carriage studies in England before and after the introduction of PCVs have shown little impact on overall carriage prevalence with the reduction in carriage of vaccine serotypes offset by an increase in non-vaccine serotypes [16]. The overall reduction in IPD cases achieved by PCVs is the consequence of the generally lower CCRs of the NVTs that replaced VTs in carriage.

Invasive pneumococcal disease is a consequence, albeit rare, of carriage of *Streptococcus pneumoniae* in the nasopharynx. Acquisition of the pneumococcus is by person-to-person transmission and occurs via respiratory droplets with transmission enhanced in settings with close mixing such as day care centres and military camps [17]. Visits to the GP for mild upper respiratory tract disease have also been shown to be a risk factor for pneumococcal acquisition [18]. It is therefore to be expected that the measures taken to reduce spread of SARS-CoV-2 would also reduce pneumococcal transmission. The impact on IPD in England and Wales of the first UK lockdown that lasted for two months from 23 March 2020 was recently reported [14]. Our model simulations of the impact of the first lockdown were similar and consistent with an overall reduction of 40% in contact patterns during this period.

Whether a reduction in coverage caused by the lockdowns would have a similar effect on other vaccine-preventable infections cannot be assessed with our model as it is specific to pneumococcal infection. However, the average number of weekly notifications of whooping cough and meningococcal septicaemia in England for one year from 23 March 2020 showed a 91.3% and 74.7% reduction respectively compared with the weekly average for the same period in 2019 [19]. As with IPD, nasopharyngeal colonisation is a pre-requisite for whooping cough and meningococcal disease. While the completeness of notifications by doctors may have suffered somewhat as a result of the COVID-19 epidemic, the magnitude of the decline in these two other infections is consistent with a substantial reduction in new colonisation episodes following the lockdown and suggests that, at least in the short term, this has outweighed any potential increase due to a reduction in coverage of pertussis or meningococcal vaccines.

Our model has limitations as previously described [10]. These include uncertainty over the future behaviour of non-vaccine serotypes and whether serotypes with different invasiveness potential will emerge, and also in the mixing patterns among the oldest age groups. However, given the magnitude of the predicted effect of the lockdowns, these uncertainties are unlikely to materially affect the results with respect to the effect of the lockdowns. It is also possible that the reduction in

For peer review only - http://bmjopen.bmj.com/site/about/guidelines.xhtml

pneumococcal carriage predicted to occur as a result of the lockdowns may affect induction of natural immunity. Nasopharyngeal carriage can boost serotype-specific IgG antibody levels to the carried serotype and such naturally induced capsular antibodies have been shown to protect against carriage of that serotype [20]. If natural immunity against carriage was reduced as a result of decreased pneumococcal transmission this could offset the reduction in IPD predicted to persist after relaxation of the lockdown. Our model has an SIS structure so does not include a natural immunity compartment, so we were unable to explore the potential effect of such perturbations in carriage-induced immunity. We were also unable to incorporate the actual coverage reductions during the two lockdown periods as coverage data for completed 1+1 PCV13 courses is only available when a birth cohort reaches two years of age. Our baseline coverage reduction scenario may therefore have been extreme with catch-up vaccination rapidly offsetting any reduction during the two lockdown periods. However, as shown by the sensitivity analysis, the critical parameter in determining impact was reductions in mixing, with only a 10% reduction having a profound effect on IPD cases.

In conclusion, our pneumococcal model predicts that the effect of the COVID-19 lockdowns on pneumococcal carriage rates will result in a reduction in IPD cases over a five year period, thus obscuring the predicted small increase in VT IPD due to the adoption of a 1+1 PCV13 schedule [10]. Pneumococcal carriage studies conducted over the next few years could help elucidate the impact of the social distancing measures and of the adoption of the 1+1 PCV13 schedule on the future incidence of VT and NVT IPD.

Contributorship statement

YC and EM conceived of the presented idea. YC an EM developed the theory and YC performed the computations. YC and EM verified the analytical methods. YC and EM discussed the results and contributed to the final manuscript.

Competing Interest: None declared

Funding: This research received no specific grant from any funding agency in the public, commercial or not-for-profit sectors.

Ethical statements

Research Ethics Approval: No ethical approval was required as the study involved no individual level data.

Consent for participation: Not required.

For peer review only - http://bmjopen.bmj.com/site/about/guidelines.xhtml

Data sharing: All the data are available in our previous publication (https://journals.plos.org/plosmedicine/article?id=10.1371/journal.pmed.1002845)

References

1 Coronavinus: First Covid-19 transmission within the UK confirmed | inews. https://inews.co.uk/news/uk/coronavirus-first-covid-19-transmission-uk-confirmed-2004500 (accessed 31 Jul 2020).

2 COVID-19: guidance on social distancing and for vulnerable people - GOV.UK. https://www.gov.uk/government/publications/covid-19-guidance-on-social-distancing-and-for-vulnerable-people (accessed 31 Jul 2020).

3 NHS. Staying at home and away from others (social distancing) - GOV.UK. https://www.gov.uk/government/publications/full-guidance-on-staying-at-home-and-away-from-others/full-guidance-on-staying-at-home-and-away-from-others (accessed 31 Jul 2020).

4 How to get medical help from home - Coronavirus (COVID-19) - NHS. https://www.nhs.uk/conditions/coronavirus-covid-19/staying-at-home-to-avoid-getting-coronavirus/how-to-get-medical-help-from-home/ (accessed 31 Jul 2020).

5 Baker C, Kirk-Wade E, Brown J, *et al.* Coronavirus: A history of English lockdown laws. https://commonslibrary.parliament.uk/research-briefings/cbp-9068/ (accessed 2 May 2021).

6 Missed vaccinations could lead to other fatal outbreaks, doctors warn | Society | The Guardian. https://www.theguardian.com/society/2020/apr/26/missed-vaccinations-could-lead-to-other-fatal-outbreaks-doctors-warn (accessed 31 Jul 2020).

7 McDonald HI, Tessier E, White JM, *et al.* Early impact of the coronavirus disease (COVID-19) pandemic and physical distancing measures on routine childhood vaccinations in England, January to April 2020. *Eurosurveillance* 2020;**25**:2000848. doi:10.2807/1560-7917.ES.2020.25.19.2000848

8 Changes to the infant pneumococcal conjugate vaccine schedule. https://assets.publishing.service.gov.uk/government/uploads/system/uploads/attachment_data/file/854153/Letter_NHSEI_PHE_PCV13_schedule_change.pdf (accessed 31 Jul 2020).

For peer review only - http://bmjopen.bmj.com/site/about/guidelines.xhtml

9 Goldblatt D, Southern J, Andrews NJ, *et al.* Pneumococcal conjugate vaccine 13 delivered as one primary and one booster dose (1 + 1) compared with two primary doses and a booster (2 + 1) in UK infants: a multicentre, parallel group randomised controlled trial. *Lancet Infect Dis* 2018;**18**:171–9. doi:10.1016/S1473-3099(17)30654-0

10 Choi YH, Andrews N, Miller E. Estimated impact of revising the 13-valent pneumococcal conjugate vaccine schedule from 2+1 to 1+1 in England and Wales: A modelling study. *PLOS Med* 2019;**16**:e1002845. doi:10.1371/journal.pmed.1002845

11 Mossong JJ, Hens N, Jit M, *et al.* Social contacts and mixing patterns relevant to the spread of infectious diseases. *PLoS Med* 2008;**5**:e74.

12 van Hoek AJ, Andrews N, Campbell H, *et al.* The social life of infants in the context of infectious disease transmission; social contacts and mixing patterns of the very young. *PLoS One.* 2013;8: 1–7. doi:10.1371/journal.pone.0076180.

13 Jarvis CI, Van Zandvoort K, Gimma A, *et al.* Quantifying the impact of physical distance measures on the transmission of COVID-19 in the UK. *BMC Med* 2020;**18**:124. doi:10.1186/s12916-020-01597-8

14 Amin-Chowdhury Z, Aiano F, Mensah A, *et al.* Impact of the Coronavirus Disease 2019 (COVID-19) Pandemic on Invasive Pneumococcal Disease and Risk of Pneumococcal Coinfection With Severe Acute Respiratory Syndrome Coronavirus 2 (SARS-CoV-2): Prospective National Cohort Study, England. *Clin Infect Dis* 2021;**72**:e65–75. doi:10.1093/cid/ciaa1728

15 Hirabayashi K. The impact of COVID-19 on routine vaccinations: Reflections during World Immunization Week 2020. https://www.unicef.org/eap/stories/impact-covid-19-routine-vaccinations (accessed 31 Jul 2020).

16 Southern J, Andrews N, Sandu P, *et al.* Pneumococcal carriage in children and their household contacts six years after introduction of the 13- valent pneumococcal conjugate vaccine in England. *PLoS One* 2018;**13**:e0195799.

17 Donkor ES. Understanding the pneumococcus: transmission and evolution. *Front Cell Infect Microbiol* 2013;**3**. doi:10.3389/FCIMB.2013.00007

18 Sleeman KL, Daniels L, Gardiner M, *et al.* Acquisition of Streptococcus pneumoniae and Nonspecific Morbidity in Infants and Their Families. *Pediatr Infect Dis J* 2005;**24**:121–7.

For peer review only - http://bmjopen.bmj.com/site/about/guidelines.xhtml

doi:10.1097/01.inf.0000151030.10159.b1

19 Notifiable diseases: weekly reports for 2020 - GOV.UK. https://www.gov.uk/government/publications/notifiable-diseases-weekly-reports-for-2020 (accessed 3 May 2021).

20 Goldblatt D, Hussain M, Andrews N, *et al.* Antibody responses to nasopharyngeal carriage of Streptococcus pneumoniae in adults: A longitudinal household study. *J Infect Dis* 2005;**192**:387–93. doi:10.1086/431524

For peer review only

For peer review only - http://bmjopen.bmj.com/site/about/guidelines.xhtml

Figure 1. Predicted annual number of IPD cases by VT and NVT and epidemiological year (July to June) from 2018/19 to 2030/31 with a 40% reduction in PCV13 coverage during the two lockdown periods starting 23 March 2020 for two months and 5 November 2020 for six months obtained from the long-term simulation model: A. Under 5 year olds and B. 65+ year olds without any reduction in contact rates: C. Under 5 year olds and D. 65+ year olds with 10% reduction in contact rates: E. Under 5 year olds and F. 65+ year olds with 40% reduction in contact rates: G. Under 5 year olds and H. 65+ year olds with 74% reduction in contact rates. Results show median and minimum and maximum of the range of model outputs generated by 500 model parameter sets [10]. (IPD, Invasive pneumococcal disease; VT, Vaccine types; NVT, Non-vaccine types)

For peer review only

Figure 2. Predicted median difference between the scenario without any change in PCV13 coverage and social mixing, and another scenario without any reduction in social mixing and no PCV13 vaccination during the two lockdowns in 2020/21 in the annual number of IPD cases, by serotype grouping (VT, NVT and overall) in all ages in England and Wales. (IPD, Invasive pneumococcal disease; VT, Vaccine types; NVT, Non-vaccine types)

For peer review only - http://bmjopen.bmj.com/site/about/guidelines.xhtml

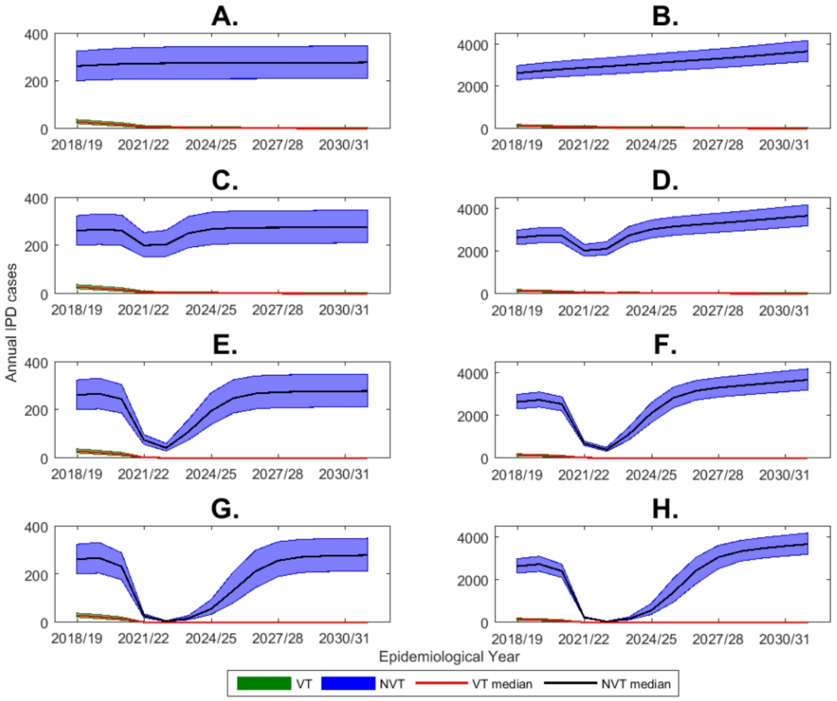

Figure 1. Predicted annual number of IPD cases by VT and NVT and epidemiological year (July to June) from 2018/19 to 2030/31 with a 40% reduction in PCV13 coverage during the two lockdown periods starting 23 March 2020 for two months and 5 November 2020 for six months obtained from the long-term simulation model: A. Under 5 year olds and B. 65+ year olds without any reduction in contact rates: C. Under 5 year olds and D. 65+ year olds with 10% reduction in contact rates: E. Under 5 year olds and F. 65+ year olds with 40% reduction in contact rates: G. Under 5 year olds and H. 65+ year olds with 74% reduction in contact rates. Results show median and minimum and maximum of the range of model outputs generated by 500 model parameter sets [10]. (IPD, Invasive pneumococcal disease; VT, Vaccine types; NVT, Non-vaccine types)

101x76mm (300 x 300 DPI)

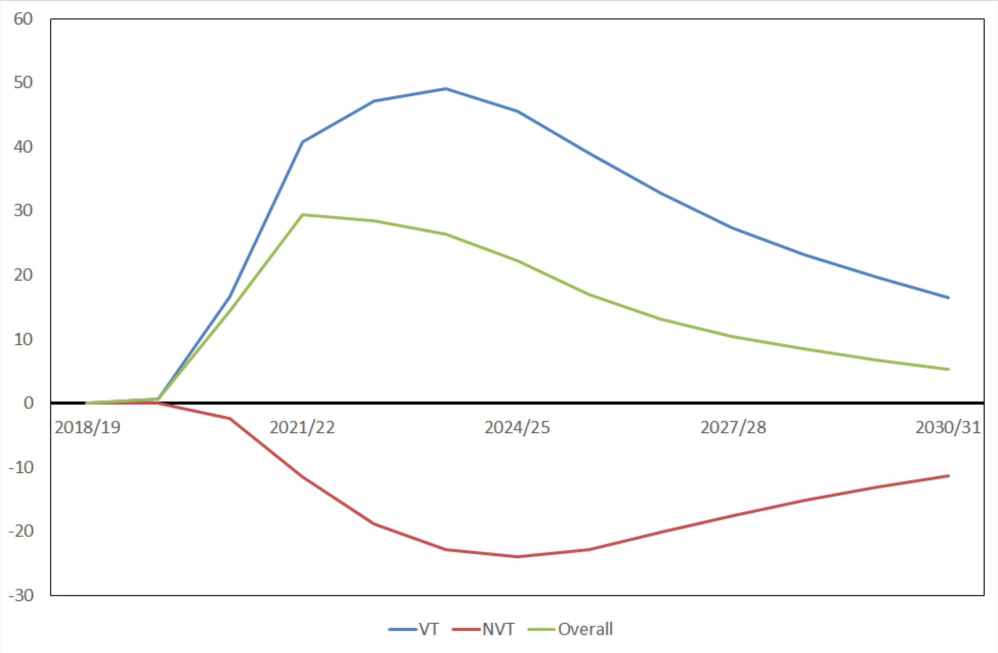

Figure 2. Predicted median difference between the scenario without any change in PCV13 coverage and social mixing, and another scenario without any reduction in social mixing and no PCV13 vaccination during the two lockdowns in 2020/21 in the annual number of IPD cases, by serotype grouping (VT, NVT and overall) in all ages in England and Wales. (IPD, Invasive pneumococcal disease; VT, Vaccine types; NVT, Non-vaccine types)

82x54mm (300 x 300 DPI)

Supplementary Appendix

Table S1. Cumulative difference in cases of invasive pneumococcal disease over 5 epidemiological years (July to June) starting from 2019/2020 by age group with different levels of reduction in PCV13 coverage and in contact rates during two lockdown periods starting 23 March for two months and 5 November 2020 for six months. Results show median and minimum and maximum of the range of model outputs generated by 500 model parameter sets [1]

Contact rate reduction	Vaccine coverage reduction	<2 yrs	2-4 yrs	5-14 yrs	15-44 yrs	45-64 yrs	65+ yrs	Total
0%	40%	3 (2, 5)	0 (0, 1)	0 (0, 1)	4 (2, 7)	4 (2, 7)	6 (2, 13)	18 (8, 33)
	100%	12 (6, 20)	2 (1, 3)	1 (1, 2)	16 (9, 26)	17 (8, 29)	25 (9, 50)	73 (33, 130)
10%	0%	-130 (-161, -97)	-47 (-57, -33)	-79 (-102, -74)	-617 (-697, -577)	-1021 (-1243, -890)	-2136 (-2484, -1837)	-4032 (-4489, -3606)
	40%	-128 (-159, -95)	-47 (-57, -33)	-79 (-102, -74)	-615 (-693, -575)	-1018 (-1240, -887)	-2130 (-2480, -1834)	-4027 (-4482, -3604)
	100%	-122 (-154, -88)	-46 (-56, -32)	-78 (-101, -73)	-607 (-685, -568)	-1009 (-1230, -880)	-2118 (-2459, -1825)	-3991 (-4432, -3566)
40%	0%	-471 (-580, -348)	-169 (-208, -118)	-275 (-349, -256)	-2062 (-2292, -1915)	-3368 (-4062, -2931)	-7107 (-8135, -6201)	-13494 (-14686, -12221)
	40%	-471 (-578, -347)	-169 (-208, -118)	-275 (-349, -256)	-2061 (-2291, -1913)	-3367 (-4062, -2931)	-7107 (-8135, -6201)	-13494 (-14676, -12211)
	100%	-468 (-576, -345)	-169 (-207, -117)	-275 (-349, -256)	-2059 (-2288, -1912)	-3366 (-4062, -2930)	-7107 (-8125, -6201)	-13484 (-14676, -12211)
74%	0%	-618 (-768, -447)	-221 (-280, -152)	-356 (-453, -329)	-2626 (-2890, -2393)	-4279 (-5183, -3690)	-9033 (-10248, -7910)	-17209 (-18511, -15541)
	40%	-617 (-768, -446)	-221 (-280, -152)	-356 (-453, -329)	-2626 (-2890, -2393)	-4279 (-5183, -3690)	-9033 (-10248, -7910)	-17209 (-18511, -15540)
	100%	-617 (-767, -446)	-221 (-280, -152)	-356 (-453, -329)	-2626 (-2890, -2392)	-4279 (-5183, -3690)	-9033 (-10248, -7910)	-17189 (-18511, -15540)

For peer review only - http://bmjopen.bmj.com/site/about/guidelines.xhtml

Table S2. Simulation results for the number of overall IPD cases between February and June 2020 and proportional reduction compared to the model predictions for the same period in 2019 (2,350 (UI 2,129, 2,531) IPD cases) in England and Wales according to different reduction levels in contact rates and vaccine coverage during two lockdowns due to COVID-19 in the UK. Results show median and minimum and maximum of the range of model outputs generated by 500 model parameter sets [1]

Contact rate reduction	Vaccine coverage reduction	IPD cases February to June 2020	Proportional reduction compared with February to June 2019
0%	40%	2,374(2,146, 2,554)	-0.9%(-1.3%, -0.6%)
	100%	2,375(2,148, 2,555)	-1.0%(-1.3%, -0.7%)
10%	0%	1,953(1,766, 2,101)	16.9%(16.5%, 17.3%)
	40%	1,954(1,766, 2,102)	16.9%(16.5%, 17.3%)
	100%	1,955(1,767, 2,103)	16.9%(16.5%, 17.2%)
40%	0%	1,125(1,017, 1,211)	52.2%(51.8%, 52.4%)
	40%	1,125(1,017, 1,211)	52.1%(51.8%, 52.4%)
	100%	1,126(1,018, 1,212)	52.1%(51.8%, 52.4%)
74%	0%	677(612, 728)	71.2%(71.1%, 71.3%)
	40%	677(612, 728)	71.2%(71.1%, 71.3%)
	100%	677(612, 729)	71.2%(71.1%, 71.3%)

For peer review only

Model description

Choi et al. [1] developed an age-structured, deterministic model structure to describe the pneumococcal transmission dynamics in England and Wales in order to estimate the potential impact of revising the 13-valent pneumococcal conjugate vaccine schedule from 2+1 to 1+1 in England and Wales. The data sources, model description, parameter estimation and fitting procedures described in this supplementary Appendix are a summary of those from Choi et al. [1]

Serotype groupings

In the model serotypes are grouped according to whether they are covered by the seven-valent pneumococcal conjugate vaccine (PCV7, VT1), or are one of the additional serotypes covered by PCV13 (VT2) or are non-vaccine serotypes (NVT). As in Choi et al [1] serotype 1 was excluded from the VT2 group as its incidence fell progressively after introduction of PCV7 in 2006 even though serotype 1 was not covered by PCV7, indicating secular changes in the behaviour of this serotype that are unrelated to vaccination. Further reductions in serotype 1 occurred after PCV13 introduction with continuing very low incidence [1] ; its exclusion from the model will therefore have a negligible impact on future IPD incidence. Serotype 3, which is also covered by the additional serotypes in PCV13, has increased progressively since 2013/14 behaving like a non-PCV13 serotype that exhibits serotype replacement. It was therefore included in the model in the NVT group [1]. For the current analysis, vaccine serotype outputs are shown combined as a single VT group though in the fitting stage VT1 and VT2 are separated to reflect the different invasiveness potential and propensity to induce serotype replacement of the VT1 and VT2 serotypes, and the differences between PCV7 and PCV13 vaccines in protecting respectively against carriage acquisition of VT1 and VT2 serotypes.

Model data

For peer review only - http://bmjopen.bmj.com/site/about/guidelines.xhtml

For peer review only

The datasets used for this modelling study include carriage prevalence data during the pre-PCV era, annual population changes by age, annual IPD cases by age groups and serogroups in pre- and post PCV introduction up to 2015/16 in England and Wales, PCV coverage data, and contact patterns in the population.

1. *Carriage prevalence and IPD incidence*

The pre-PCV7 carriage prevalence in seven age groups (under 1, 1-2, 3-4, 5-9, 10-19, 20-39 and 40+ years) and the three serotype groupings VT1, VT2 and NVT was obtained from a longitudinal family study [2] in which 3,869 nasopharyngeal swabs were taken from 489 individuals who were swabbed monthly over ten months between 2001 and 2002 in England.

The national enhanced surveillance dataset of serotyped IPD cases collated by Public Health England for epidemiological years (July to June) between 2000/01 and 2005/6 was used for the pre-PCV7 baseline incidence and the epidemiological years from 2005/6 to 2015/16 as the post-PCV period for the purposes of model fitting. The carriage prevalence and IPD datasets are available at https://doi.org/10.1371/journal.pmed.1002845.s001.

2. *Population structure and contact patterns*

The annual population size by age in each year from the pre-PCV7 to the post-PCV13 period was obtained from census data and the estimated demographic changes out to 2030 from the Office of National Statistics [3]. The contact pattern within and between age groups was derived from the POLYMOD survey conducted in the UK in 2006 [4] supplemented by an additional contact survey among infants under one year [5].

3. *PCV coverage data*

Vaccine coverage by dose, monthly birth cohort and calendar month in the PCV7 catch-up and the routine 2+1 PCV7 programme up to 2008 was obtained from the General Practice Research database

For peer review only - http://bmjopen.bmj.com/site/about/guidelines.xhtml

as previously described [6]. Thereafter, coverage for the second priming dose and the booster dose was obtained from annual national coverage data [7].

Model structure

1. ***Model population***

The population in the model is divided into 100 annual age cohorts (0, 1, 2, 3, … , 99). Each annual age-cohort is divided into 48 equal-sized age-cohorts (in total 4,800 age-cohorts in the total population in the model).

2. ***Transition between model compartments***

In the absence of vaccination individuals are born susceptible (S) to pneumococcal carriage and become infected (I) with a VT1, VT2 or NVT serotype as determined by the serogroup-specific force of infection. An episode of carriage does not result in protection against subsequent carriage of any serotype (i.e. SIS model structure). Invasive disease is assumed to occur at the time of carriage acquisition whereas transmission can occur at any time during the carriage episode. Individuals clear their infection with age-dependent clearance rates as estimated previously [8] and become susceptible again. Individuals already carrying a serotype from one of the three groupings will have some degree of protection against infections from another serotype grouping according to the level of competition between the three serotype groupings. These competition parameters determine the extent of serotype replacement in carriage (and therefore in IPD) when vaccine serotypes decline post-PCV introduction. The flowchart for the model structure is available at https://doi.org/10.1371/journal.pmed.1002845.g002.

3. ***Vaccine efficacy parameters***

Within the vaccine protected group, two doses in the first year of life or one dose after 12 months of age are assumed to confer the maximum degree of protection that can be obtained from PCV7 or

For peer review only - http://bmjopen.bmj.com/site/about/guidelines.xhtml

PCV13 against carriage acquisition of the respective serotype grouping (termed full protection). A single dose in the first year of life is assumed to provide half the maximum protection (termed partial protection). Protection wanes exponentially with fully protected individuals moving back to the partially protected group and then back to the fully susceptible group. For the base case the average duration of protection for both full and partial protection is set at 5 years.

Vaccine efficacy against IPD given carriage of a vaccine serotype for fully protected individuals is assumed to be 100% at the time of vaccination and to wane with the same average duration as for the full and partial protection respectively against carriage. The flowchart showing the transitions between the unvaccinated, partially protected and fully protected compartments as a result of vaccination and subsequent waning is available at https://doi.org/10.1371/journal.pmed.1002845.g003.

The propensity to develop invasive disease upon carriage acquisition within each age group will be determined by the overall case-carrier ratio (CCR) of serotypes comprising the VT1, VT2 and NVT groupings (https://doi.org/10.1371/journal.pmed.1002845.g006) and is derived from the ratio of IPD cases: incident carriage infections within each serotype grouping in each age group.

Model parameter estimation

The model fitting process consists of a static pre-vaccination equilibrium component and post-vaccination dynamic component. By fitting the model to the given datasets, we estimate 26 model parameters: competition parameters for six age groups and three serotype groupings, the two vaccine efficacies against carriage acquisition of VT1 and VT2 serotypes and an additional parameter that allows for an increase in the case carrier ratio of the serotypes in the NVT group after 2013/14 when the rate of serotype replacement suddenly increased [9]. This last parameter is allowed to vary between each of the six age groups generating in total 26 parameters. A summary

For peer review only - http://bmjopen.bmj.com/site/about/guidelines.xhtml

table showing the derivations of the model parameters is available at https://journals.plos.org/plosmedicine/article/figure?id=10.1371/journal.pmed.1002845.t001.

As described in Choi et al [1] the force of infection and clearance parameters that determine the rate of acquisition and termination of a new carriage episode for the VT1, VT2 and NVT groups were estimated by fitting a static model to the pre-vaccination carriage data. The competition parameters that determine the extent to which carriage of serotypes in one group protects against acquisition of serotypes in another group were estimated by fitting a post-vaccination model to the changes in serotype grouping after the introduction of PCV7 and PCV13 from 2005/6 to 2015/16.

The uncertainty intervals were generated from 500 randomly selected sets of model parameters that generated outputs within ±0.3 of the set that gave the maximum likelihood value obtained using the Nelder-Mead Downhill Simplex method [10] when compared with the post-PCV IPD data. Results are shown as the median value of the accepted 500 parameters sets and the minimum to maximum range is shown as the uncertainty interval (UI) at https://journals.plos.org/plosmedicine/article/figure?id=10.1371/journal.pmed.1002845.t002. VEcVT1 (Vaccine efficacy against acquiring VT1 carriage) and VEcVT2 (Vaccine efficacy against acquiring VT2 carriage) were estimated at 0.55 (0.53, 0.57) and 0.30 (0.26, 0.36) respectively (minimum and maximum).

Long-term simulations

Using the 500 parameter sets obtained for the uncertainty boundary the model simulated various scenarios to investigate the potential impact of two lockdowns occurred during the COVID-19 pandemic until 2030/31, the furthest year for which there are population age-structure predictions.

References

1 Choi YH, Andrews N, Miller E. Estimated impact of revising the 13-valent pneumococcal conjugate vaccine schedule from 2+1 to 1+1 in England and Wales: A modelling study. *PLoS*

For peer review only - http://bmjopen.bmj.com/site/about/guidelines.xhtml

Med 2019;**16**. doi:10.1371/journal.pmed.1002845

2 Hussain M, Melegaro A, Pebody RG, *et al.* A longitudinal household study of Streptococcus pneumoniae nasopharyngeal carriage in a UK setting. *Epidemiol Infect* 2005;**133**:891–8.

3 Office for National Statistics. England Wales population size. https://www.ons.gov.uk/

4 Mossong JJ, Hens N, Jit M, *et al.* Social contacts and mixing patterns relevant to the spread of infectious diseases. *PLoS Med* 2008;**5**:e74.

5 van Hoek AJ, Andrews N, Campbell H, *et al.* The social life of infants in the context of infectious disease transmission; social contacts and mixing patterns of the very young. *PLoS One* 2013;**8**:e76180.http://www.pubmedcentral.nih.gov/articlerender.fcgi?artid=3797797&tool=pmcentrez&rendertype=abstract (accessed 30 Sep 2014).

6 Choi YH, Jit M, Gay N, *et al.* 7-Valent Pneumococcal Conjugate Vaccination in England and Wales: Is It Still Beneficial Despite High Levels of Serotype Replacement? *PLoS One* 2011;**6**:e26190.

7 Public Health England. Vaccine uptake guidance and the latest coverage data. 2018.https://www.gov.uk/government/collections/vaccine-uptake

8 Melegaro A, Gay NJ, Medley GF, *et al.* Estimating the transmission parameters of pneumococcal carriage in households. *Epidemiol Infect* 2004;**132**:433–41.

9 Ladhani SN, Collins S, Djennad A, *et al.* Rapid increase in non-vaccine serotypes causing invasive pneumococcal disease in England and Wales, 2000-17: a prospective national observational cohort study. *Lancet Infect Dis* 2018;**18**:441–51.

10 Nelder JA, Mead R. A Simplex Method for Function Minimization. *Comput J* 1965;**7**:308–13. doi:10.1093/comjnl/7.4.308

For peer review only

For peer review only - http://bmjopen.bmj.com/site/about/guidelines.xhtml

BMJ Open

Impact of Covid-19 social distancing measures on future incidence of invasive pneumococcal disease in England and Wales – a mathematical modelling study

Journal:	*BMJ Open*
Manuscript ID	bmjopen-2020-045380.R2
Article Type:	Original research
Date Submitted by the Author:	31-Aug-2021
Complete List of Authors:	Choi, Yoon Hong; Public Health England, Statistics, Modelling and Economics Department<br>Miller, Elizabeth; London School of Hygiene & Tropical Medicine
**Primary Subject Heading**:	Public health
Secondary Subject Heading:	Epidemiology, Infectious diseases, Qualitative research
Keywords:	COVID-19, PUBLIC HEALTH, INFECTIOUS DISEASES, EPIDEMIOLOGY

SCHOLARONE™
Manuscripts

I, the Submitting Author has the right to grant and does grant on behalf of all authors of the Work (as defined in the below author licence), an exclusive licence and/or a non-exclusive licence for contributions from authors who are: i) UK Crown employees; ii) where BMJ has agreed a CC-BY licence shall apply, and/or iii) in accordance with the terms applicable for US Federal Government officers or employees acting as part of their official duties; on a worldwide, perpetual, irrevocable, royalty-free basis to BMJ Publishing Group Ltd ("BMJ") its licensees and where the relevant Journal is co-owned by BMJ to the co-owners of the Journal, to publish the Work in this journal and any other BMJ products and to exploit all rights, as set out in our licence.

The Submitting Author accepts and understands that any supply made under these terms is made by BMJ to the Submitting Author unless you are acting as an employee on behalf of your employer or a postgraduate student of an affiliated institution which is paying any applicable article publishing charge ("APC") for Open Access articles. Where the Submitting Author wishes to make the Work available on an Open Access basis (and intends to pay the relevant APC), the terms of reuse of such Open Access shall be governed by a Creative Commons licence – details of these licences and which Creative Commons licence will apply to this Work are set out in our licence referred to above.

Other than as permitted in any relevant BMJ Author's Self Archiving Policies, I confirm this Work has not been accepted for publication elsewhere, is not being considered for publication elsewhere and does not duplicate material already published. I confirm all authors consent to publication of this Work and authorise the granting of this licence.

For peer review only

Impact of Covid-19 social distancing measures on future incidence of invasive pneumococcal disease in England and Wales – a mathematical modelling study

Yoon Hong Choi[1*] and Elizabeth Miller[2]

1. Statistics and Modelling Economics Department, Public Health England, London, England

2. Department of Infectious Disease Epidemiology, Faculty of Epidemiology & Population Health, London School of Hygiene & Tropical Medicine, London, England

* Corresponding author

Address: Modelling and Economics Unit, Statistics and Modelling Economics Department, Public Health England, 61 Colindale Ave, London, NW9 5EQ, United Kingdom

E-mail: Yoon.choi@phe.gov.uk

Tel: +44 (0) 20 8327 7454

Fax: +44 (0) 20 8200 4400

Key words: COVID-19, Social distancing, Lockdown, Pneumococcal transmission model, Invasive pneumococcal disease, Pneumococcal Conjugate Vaccine

Abstract

Objectives

In January 2020, the United Kingdom moved to a 1+1 schedule for the 13-valent pneumococcal conjugate vaccine (PCV13) with a single priming dose at 3 months and a 12month booster. We modelled the impact on invasive pneumococcal disease (IPD) out to 2030/31 of reductions in PCV13 coverage and population mixing associated with restrictions on non-essential health care visits and social distancing measures introduced in 2020/21 to reduce SARS-CoV-2 transmission.

Design

Using an existing model of pneumococcal transmission in England and Wales we simulated the impact of a 40% reduction in coverage and a 40% reduction in mixing between and within age-

For peer review only - http://bmjopen.bmj.com/site/about/guidelines.xhtml

For peer review only

groups during two lockdowns in spring 2020 and autumn/winter 2020/21. More and less extreme reductions in coverage and mixing were explored in a sensitivity analysis.

Main outcome measures

Predicted annual numbers of IPD cases under different coverage and mixing reduction scenarios with uncertainty intervals (UI) generated from minimum and maximum values of the model predictions using 500 parameter sets.

Results

The model predicted that any increase in IPD cases resulting from a reduction in PCV13 coverage would be more than offset by a reduction in pneumococcal transmission due to social distancing measures and that overall reductions in IPD cases will persist for a few years after resumption of normal mixing. The net reduction in cumulative IPD cases over the five epidemiological years from July 2019 was predicted to be 13,494 (UI 12,211, 14,676) all ages. Similar results were obtained in the sensitivity analysis.

Conclusion

COVID-19 lockdowns are predicted to have had a profound effect on pneumococcal transmission resulting in a reduction in pneumococcal carriage prevalence and IPD incidence for up to five years after the end of the lockdown period. Carriage studies will be informative in confirming the predicted impact of the lockdown measures after they have been lifted.

Strengths and limitations of this study

For this study we used a previously published dynamic model of pneumococcal transmission that was fitted to past data on age-specific pneumococcal carriage and IPD and was able to reproduce the observed herd immunity effects of the pneumococcal conjugate vaccination programme in England and Wales. Short term model predictions of the effect of the first lockdown were validated against IPD data in England and Wales to June 2020.

Our conclusions were robust under the extreme scenario of assuming only a 10% reduction in mixing as a result of the two lockdowns and suspension of all PCV13 vaccination during the lockdown periods with no subsequent catch-up vaccination.

A limitation of our model is uncertainty over the future behaviour of non-vaccine serotypes although this is unlikely to affect the overall conclusions.

For peer review only - http://bmjopen.bmj.com/site/about/guidelines.xhtml

The predicted effect of the reduction in social mixing on carriage of pneumococci could potentially reduce development of natural immunity as a result of carriage but we were unable with the current model structure to investigate this.

For peer review only

For peer review only - http://bmjopen.bmj.com/site/about/guidelines.xhtml

Introduction

Following the first identification of local transmission of SARS-CoV-2 in England on February 28, 2020, [1] the United Kingdom (UK) government instituted a series of control measures to reduce spread of the virus. On March 18 schools along with restaurants and places of entertainment and leisure were closed, and on March 23 all non-essential travel was banned with members of the population only permitted to leave their home for exercise once a day, or in order to obtain food or medication [2]. The public was advised to only use the National Health Service when essential [3] and many general practices replaced face-to-face consultations with an online service [4]. This first national lockdown was eased in stages starting in May 2020 with children returning to schools in September 2020 [5]. As COVID-19 cases started to increase again in September 2020, a variety of locally-based measures were introduced in areas of increasing incidence in an attempt to reduce transmission. Despite these local measures, cases continued to increase and a second national lockdown came into force in November 2020 which lasted for six months until May 2021 [5].

In the UK, paediatric vaccinations are given in general practice and there was initial concern that the measures taken to limit spread of SARS-CoV-2 would result in many children's vaccinations being postponed with a resulting increase in vaccine-preventable infections [6]. Indeed early coverage data for measles/mumps/rubella vaccine for the first 3 weeks of the first lockdown in London suggested a drop of over 40% compared with the same period in 2019 [7]. Of particular concern is the impact of a drop in coverage of the 13 valent pneumococcal conjugate vaccine (PCV13) for which the national 2+1 schedule (2, 4, and 12 months) was changed on 1 January 2020 to a 1+1 schedule at 3 and 12 months [8]. The rationale for the schedule change was that after a decade of high coverage with PCV13 much of the protection of young children against vaccine-type invasive pneumococcal disease (IPD) is through herd immunity generated by the 12 month booster dose and that dropping a priming dose would have little effect on this. The supporting evidence for the schedule change came from an immunogenicity study showing similar post-booster antibody responses to PCV13 serotypes with a 2+1 and 1+1 schedule [9] and a modelling study which predicted little impact on IPD cases in children or adults if protection against vaccine-type carriage was similar post-booster with the two schedules [10].

Using our previously described pneumococcal transmission model [10] we investigated the impact on IPD if PCV13 coverage was substantially reduced during the lockdown periods in the UK. The model also allowed us to investigate the likely longer-term impact on pneumococcal transmission of social mixing restrictions put in place to limit the spread of the SARS-CoV-2 virus in England and Wales.

For peer review only - http://bmjopen.bmj.com/site/about/guidelines.xhtml

Model and assumptions.

The same compartmental deterministic model and estimated parameter values as described by Choi et al. [10] were used in this study. Briefly, the model simulates the transmission dynamics of carried pneumococci between and within different age groups and predicts carriage changes by serotype grouping, either vaccine type (VT) or non-vaccine type (NVT), under different vaccination scenarios. Carriage changes are then translated into IPD cases using case-carrier ratios (CCRs) that describe the invasive potential of different carried serotypes. The model has a Susceptible-Infected-Susceptible (SIS) structure as it is assumed that a carriage episode does not generate any serogroup-specific protection. The model has 26 parameters which describe the propensity for vaccine-induced replacement of VTs with NVTs in carriage, the CCRs in different age groups, and the effectiveness of PCV vaccination against carriage of different vaccine serotypes. (see Supplementary Appendix for summary of model description and parameter estimation). The maximum likelihood values of these parameters and their uncertainty intervals are estimated by fitting the model to IPD data up to the end of the epidemiological year from July 2015 to June 2016 [10]. In implementing the effect of the 1+1 schedule, the base case in the model described by Choi et al. [10] assumed that one priming dose at 3 months of age provides half the protection against VT carriage as two doses while the effect of the 12 month booster remains unchanged. In the current analysis, the change to the 1+1 PCV13 schedule was implemented to reflect the actual start date of January 2020, not the assumed date of September 2018 as in the original model [10].

We assumed that, during the first two-month lockdown period starting from 23rd March 2020 and also during the second six-month lockdown starting from 5th November 2020, there was a 40% reduction in PCV13 coverage, as suggested by early declines in MMR vaccine coverage in London during the first lockdown [7]. For the sensitivity analysis we assumed two additional scenarios, one with no material decline in PCV13 coverage (0% decline) and a second with cessation of all PCV13 vaccination during the lockdown periods (100% decline). The 0% scenario was chosen to reflect a situation in which there was a rapid catch-up of missed PCV13 vaccinations after the first lockdown with no reduction during the second while the 100% reduction scenario was explored as an extreme scenario. We pessimistically assumed there would be no subsequent catch up vaccination for children under the 40% and 100% reduction scenarios as final coverage data for the affected birth cohorts will not be available until 2021/22.

To implement the impact of reduced social mixing during each of the lockdown periods we used the same mixing matrix as before [10] based on the POLYMOD study [11] and an additional contact survey among infants under one year [12] but reduced the number of contacts between and within

For peer review only - http://bmjopen.bmj.com/site/about/guidelines.xhtml

each age group by 40% as a baseline scenario and explored 10% and 74% reduction scenarios for the sensitivity analysis. The 74% reduction scenario was based on early data from a study of the social contacts of 1,356 UK adults between 24 and 27 March [13] compared with the historical rates documented in the POLYMOD study [11]. However, this study [13] could not assess the impact on contacts made by children, particularly those of pre-school age who are the main drivers of pneumococcal transmission [10]. As the social distancing measures in children were less strict than adults, the estimated reduction in mixing would be lower in children and so we took the 74% scenario as the upper extreme scenario for the contact pattern reduction.

Results are shown as the median of the model outputs under the different scenarios. The uncertainty interval (UI) for each scenario represents the minimum and maximum values generated with 500 model parameters sets obtained from Choi et al. [10]. The impact on IPD of the temporary changes in PCV13 coverage and social mixing is presented by VT, which includes PCV13 serotypes minus serotypes 1 and 3, and NVT; serotype 3 is included with the NVT group in the model on the grounds that it has shown an increase post-PCV13 similar to that seem for NVTs while serotype 1 is excluded from the model due to secular changes which are independent of vaccine usage [10] .

Patient and Public Involvement statement: Our study did not involve any direct participation by human subjects.

Results

Fig 1 shows the predicted number of IPD cases in England and Wales by epidemiological year out to 2030/31 in under 5 year olds and in 65+ year olds by VT and NVT group with four different scenarios. The first scenario presented in Figs 1 A&B is without any change in PCV13 coverage and contact patterns; the increase in IPD cases out to 2030/31 in 65+ year olds reflects the increasing mean age of individuals in this age group in the future together with the steep increase in IPD incidence with increasing age in the elderly population. The remaining three scenarios in Figs 1 C-H assume a 40% reduction in PCV13 coverage with respectively a 10% (C&D), 40% (E&F) or 74% (G&H) reduction in contact rates during the two lockdowns. Even with only a 10% reduction in contact rates in Figs 1 C&D, the predicted impact of reduced social mixing outweighed any increase in IPD due to reduction in PCV13 coverage. The predicted beneficial effect of the lockdown on IPD cases was even more marked with higher reductions in social mixing (Figs 1 E, F, G and H). Under all the scenarios, the model predicted that the impact of the two lockdowns on IPD would persist for up to five years after their cessation.

For peer review only - http://bmjopen.bmj.com/site/about/guidelines.xhtml

Table 1. Cumulative difference in cases of invasive pneumococcal disease over 5 epidemiological years (July to June) starting from 2019/2020 by age group and serotype grouping (VT, NVT and overall) with a 40% reduction in PCV13 coverage, 40% reduction in contact rates during the two lockdown periods starting from 23 March for two months and from 5 November 2020 for six months. Results show median and minimum and maximum of the range of model outputs generated by 500 model parameter sets [10]. (VT, Vaccine types; NVT, Non-vaccine types)

	0-1Y	2-4Y	5-14Y	15-44Y	45-64Y	65Y+	Overall
VT	-13 (-26, -6)	-2 (-4, -1)	-8 (-15, -3)	-82 (-164, -36)	-94 (-186, -41)	-156 (-312, -69)	-354 (-707, -156)
NVT	-456 (-564, -334)	-167 (-206, -116)	-268 (-343, -249)	-1977 (-2195, -1836)	-3282 (-3976, -2844)	-6938 (-7956, -6053)	-13123 (-14275, -11893)
Overall	-471 (-578, -347)	-169 (-208, -118)	-275 (-349, -256)	-2061 (-2291, -1913)	-3367 (-4062, -2931)	-7107 (-8135, -6201)	-13494 (-14676, -12211)

The cumulative number of additional IPD cases predicted in England and Wales over the first five epidemiological years starting from July 2019 is shown in Table 1 by age group and serotype grouping for a 40% reduction in contact rates and 40% reduction in PCV13 coverage during two lockdowns (base case scenario). Across all ages the model predicted a net saving of 13,494 IPD cases (UI 12,211, 14,676) over this period compared with the predicted number of IPD cases without any lockdowns. The results over the first five years by age group and different scenarios for the reduction in coverage and social mixing are presented in Table S1. Without any reduction in contact rates under the worst case of no PCV13 vaccination during the two lockdown periods and no catch up vaccination, the model predicted a cumulative additional 105 (UI 49, 197) VT IPD cases, of which 14 (UI 6, 26) were in children under 2 years of age over the first five epidemiological years since 2019/20. The overall predicted increase in IPD cases was smaller (73 (UI 33, 130)) due to a concomitant reduction in replacement with NVT IPD cases (Fig 2). While the predicted overall increase is small the perturbation in VT IPD cases as a result of the temporary interruption of vaccination is predicted to persist out to 2030/31.

Amin-Chowdbury et al. [14] reported that there were 2,440 IPD cases in England and Wales between February and June 2019 and 1,137 IPD cases during the same period in 2020 showing a 53%

For peer review only - http://bmjopen.bmj.com/site/about/guidelines.xhtml

reduction in overall IPD cases in England and Wales. The model predicted that there would be 2,350 (UI 2,129, 2,531) IPD cases in the same period in 2019.

Table 2 shows the model predictions for the reduction in IPD cases in February to June 2020 with a 40% reduction in PCV13 coverage during the two lockdown periods and under various reduced mixing scenarios. The scenario with a 40% reduction in mixing pattern most closely matched the observed reduction in IPD during February to June 2020 [14]. Table S2 shows the predicted number of IPD cases during February to June 2020 with different assumptions about reductions in PCV13 coverage and in social mixing. Only the scenarios with a 40% reduction in mixing produced the observed reductions seen in IPD incidence with little impact of varying the PCV13 coverage reductions.

For peer review only

Table 2. Model outputs for the number of overall IPD cases between February and June 2020 and proportional reduction compared to the model predictions for the same period in 2019 (2,350 (UI 2,129, 2,531) IPD cases) in England and Wales according to different reduction levels in contact patterns with 40% PCV13 coverage reduction rate during the first two lockdown starting 23 March for 2 months in the UK. Results show median and minimum and maximum of the range of model outputs generated by 500 model parameter sets [10]. (IPD, Invasive pneumococcal disease)

Reduction in mixing	2020	Proportional reduction
0%	2,374(2,146, 2,554)	-0.9%(-1.3%, -0.6%)
10%	1,954(1,766, 2,102)	16.9%(16.5%, 17.3%)
40%	1,125(1,017, 1,211)	52.1%(51.8%, 52.4%)
74%	677(612, 728)	71.2%(71.1%, 71.3%)

Discussion

In line with many other countries, the UK adopted a lockdown strategy to minimise social contacts and thereby reduce the spread of the SARS-CoV-2. The restriction on non-essential travel and GP visits raised concerns that vaccine coverage in children might be substantially reduced [6], echoing similar concerns raised at the global level [15]. With the recent change to a 1+1 PCV13 schedule in the UK, which is reliant on the booster dose to maintain herd immunity [9,10], a substantial reduction in PCV13 coverage could compromise control of IPD. Our modelling study suggests that any potential impact of the lockdown measures on PCV13 coverage and herd immunity will be more than offset by a reduction in pneumococcal transmission due to the reduction in population mixing such that a net reduction in IPD cases should occur. Our model also predicts that the net reduction in

For peer review only - http://bmjopen.bmj.com/site/about/guidelines.xhtml

IPD cases will be sustained for up to five years after mixing has returned to normal levels in the population. This reduction in IPD would be the result of an overall reduction in pneumococcal carriage prevalence after the two lockdown periods, unlike the IPD reduction achieved with PCV. Carriage studies in England before and after the introduction of PCVs have shown little impact on overall carriage prevalence with the reduction in carriage of vaccine serotypes offset by an increase in non-vaccine serotypes [16]. The overall reduction in IPD cases achieved by PCVs is the consequence of the generally lower CCRs of the NVTs that replaced VTs in carriage.

Invasive pneumococcal disease is a consequence, albeit rare, of carriage of *Streptococcus pneumoniae* in the nasopharynx. Acquisition of the pneumococcus is by person-to-person transmission and occurs via respiratory droplets with transmission enhanced in settings with close mixing such as day care centres and military camps [17]. Visits to the GP for mild upper respiratory tract disease have also been shown to be a risk factor for pneumococcal acquisition [18]. It is therefore to be expected that the measures taken to reduce spread of SARS-CoV-2 would also reduce pneumococcal transmission. The impact on IPD in England and Wales of the first UK lockdown that lasted for two months from 23 March 2020 was recently reported [14]. Our model simulations of the impact of the first lockdown were similar and consistent with an overall reduction of 40% in contact patterns during this period.

Whether a reduction in coverage caused by the lockdowns would have a similar effect on other vaccine-preventable infections cannot be assessed with our model as it is specific to pneumococcal infection. However, the average number of weekly notifications of whooping cough and meningococcal septicaemia in England for one year from 23 March 2020 showed a 91.3% and 74.7% reduction respectively compared with the weekly average for the same period in 2019 [19]. As with IPD, nasopharyngeal colonisation is a pre-requisite for whooping cough and meningococcal disease. While the completeness of notifications by doctors may have suffered somewhat as a result of the COVID-19 epidemic, the magnitude of the decline in these two other infections is consistent with a substantial reduction in new colonisation episodes following the lockdown and suggests that, at least in the short term, this has outweighed any potential increase due to a reduction in coverage of pertussis or meningococcal vaccines.

Our model has limitations as previously described [10]. These include uncertainty over the future behaviour of non-vaccine serotypes and whether serotypes with different invasiveness potential will emerge, and also in the mixing patterns among the oldest age groups. However, given the magnitude of the predicted effect of the lockdowns, these uncertainties are unlikely to materially affect the results with respect to the effect of the lockdowns. It is also possible that the reduction in

For peer review only - http://bmjopen.bmj.com/site/about/guidelines.xhtml

pneumococcal carriage predicted to occur as a result of the lockdowns may affect induction of natural immunity. Nasopharyngeal carriage can boost serotype-specific IgG antibody levels to the carried serotype and such naturally induced capsular antibodies have been shown to protect against carriage of that serotype [20]. If natural immunity against carriage was reduced as a result of decreased pneumococcal transmission this could offset the reduction in IPD predicted to persist after relaxation of the lockdown. Our model has an SIS structure so does not include a natural immunity compartment, so we were unable to explore the potential effect of such perturbations in carriage-induced immunity. We were also unable to incorporate the actual coverage reductions during the two lockdown periods as coverage data for completed 1+1 PCV13 courses is only available when a birth cohort reaches two years of age. Our baseline coverage reduction scenario may therefore have been extreme with catch-up vaccination rapidly offsetting any reduction during the two lockdown periods. However, as shown by the sensitivity analysis, the critical parameter in determining impact was reductions in mixing, with only a 10% reduction having a profound effect on IPD cases.

In conclusion, our pneumococcal model predicts that the effect of the COVID-19 lockdowns on pneumococcal carriage rates will result in a reduction in IPD cases over a five year period, thus obscuring the predicted small increase in VT IPD due to the adoption of a 1+1 PCV13 schedule [10]. Pneumococcal carriage studies conducted over the next few years could help elucidate the impact of the social distancing measures and of the adoption of the 1+1 PCV13 schedule on the future incidence of VT and NVT IPD.

Contributorship statement

YC and EM conceived of the presented idea. YC an EM developed the theory and YC performed the computations. YC and EM verified the analytical methods. YC and EM discussed the results and contributed to the final manuscript.

Competing Interest: None declared

Funding: Elizabeth Miller is funded by the National Institute for Health Research (NIHR) Health Protection Research Unit in Immunisation at the London School of Hygiene and Tropical Medicine in partnership with Public Health England (**Grant Reference NIHR200929**).

Ethical statements

Research Ethics Approval: No ethical approval was required as the study involved no individual level data.

For peer review only - http://bmjopen.bmj.com/site/about/guidelines.xhtml

Consent for participation: Not required.

Data sharing: All the data are available in our previous publication (https://journals.plos.org/plosmedicine/article?id=10.1371/journal.pmed.1002845)

References

1 Coronavinus: First Covid-19 transmission within the UK confirmed | inews. https://inews.co.uk/news/uk/coronavirus-first-covid-19-transmission-uk-confirmed-2004500 (accessed 31 Jul 2020).

2 COVID-19: guidance on social distancing and for vulnerable people - GOV.UK. https://www.gov.uk/government/publications/covid-19-guidance-on-social-distancing-and-for-vulnerable-people (accessed 31 Jul 2020).

3 NHS. Staying at home and away from others (social distancing) - GOV.UK. https://www.gov.uk/government/publications/full-guidance-on-staying-at-home-and-away-from-others/full-guidance-on-staying-at-home-and-away-from-others (accessed 31 Jul 2020).

4 How to get medical help from home - Coronavirus (COVID-19) - NHS. https://www.nhs.uk/conditions/coronavirus-covid-19/staying-at-home-to-avoid-getting-coronavirus/how-to-get-medical-help-from-home/ (accessed 31 Jul 2020).

5 Baker C, Kirk-Wade E, Brown J, *et al.* Coronavirus: A history of English lockdown laws. https://commonslibrary.parliament.uk/research-briefings/cbp-9068/ (accessed 2 May 2021).

6 Missed vaccinations could lead to other fatal outbreaks, doctors warn | Society | The Guardian. https://www.theguardian.com/society/2020/apr/26/missed-vaccinations-could-lead-to-other-fatal-outbreaks-doctors-warn (accessed 31 Jul 2020).

7 McDonald HI, Tessier E, White JM, *et al.* Early impact of the coronavirus disease (COVID-19) pandemic and physical distancing measures on routine childhood vaccinations in England, January to April 2020. *Eurosurveillance* 2020;**25**:2000848. doi:10.2807/1560-7917.ES.2020.25.19.2000848

8 Changes to the infant pneumococcal conjugate vaccine schedule. https://assets.publishing.service.gov.uk/government/uploads/system/uploads/attachment_d

For peer review only - http://bmjopen.bmj.com/site/about/guidelines.xhtml

ata/file/854153/Letter_NHSEI_PHE_PCV13_schedule_change.pdf (accessed 31 Jul 2020).

9 Goldblatt D, Southern J, Andrews NJ, *et al.* Pneumococcal conjugate vaccine 13 delivered as one primary and one booster dose (1 + 1) compared with two primary doses and a booster (2 + 1) in UK infants: a multicentre, parallel group randomised controlled trial. *Lancet Infect Dis* 2018;**18**:171–9. doi:10.1016/S1473-3099(17)30654-0

10 Choi YH, Andrews N, Miller E. Estimated impact of revising the 13-valent pneumococcal conjugate vaccine schedule from 2+1 to 1+1 in England and Wales: A modelling study. *PLOS Med* 2019;**16**:e1002845. doi:10.1371/journal.pmed.1002845

11 Mossong JJ, Hens N, Jit M, *et al.* Social contacts and mixing patterns relevant to the spread of infectious diseases. *PLoS Med* 2008;**5**:e74.

12 van Hoek AJ, Andrews N, Campbell H, *et al.* The social life of infants in the context of infectious disease transmission; social contacts and mixing patterns of the very young. *PLoS One.* 2013;8: 1–7. doi:10.1371/journal.pone.0076180.

13 Jarvis CI, Van Zandvoort K, Gimma A, *et al.* Quantifying the impact of physical distance measures on the transmission of COVID-19 in the UK. *BMC Med* 2020;**18**:124. doi:10.1186/s12916-020-01597-8

14 Amin-Chowdhury Z, Aiano F, Mensah A, *et al.* Impact of the Coronavirus Disease 2019 (COVID-19) Pandemic on Invasive Pneumococcal Disease and Risk of Pneumococcal Coinfection With Severe Acute Respiratory Syndrome Coronavirus 2 (SARS-CoV-2): Prospective National Cohort Study, England. *Clin Infect Dis* 2021;**72**:e65–75. doi:10.1093/cid/ciaa1728

15 Hirabayashi K. The impact of COVID-19 on routine vaccinations: Reflections during World Immunization Week 2020. https://www.unicef.org/eap/stories/impact-covid-19-routine-vaccinations (accessed 31 Jul 2020).

16 Southern J, Andrews N, Sandu P, *et al.* Pneumococcal carriage in children and their household contacts six years after introduction of the 13- valent pneumococcal conjugate vaccine in England. *PLoS One* 2018;**13**:e0195799.

17 Donkor ES. Understanding the pneumococcus: transmission and evolution. *Front Cell Infect Microbiol* 2013;**3**. doi:10.3389/FCIMB.2013.00007

18 Sleeman KL, Daniels L, Gardiner M, *et al.* Acquisition of Streptococcus pneumoniae and

For peer review only - http://bmjopen.bmj.com/site/about/guidelines.xhtml

Nonspecific Morbidity in Infants and Their Families. *Pediatr Infect Dis J* 2005;**24**:121–7. doi:10.1097/01.inf.0000151030.10159.b1

19 Notifiable diseases: weekly reports for 2020 - GOV.UK. https://www.gov.uk/government/publications/notifiable-diseases-weekly-reports-for-2020 (accessed 3 May 2021).

20 Goldblatt D, Hussain M, Andrews N, *et al.* Antibody responses to nasopharyngeal carriage of Streptococcus pneumoniae in adults: A longitudinal household study. *J Infect Dis* 2005;**192**:387–93. doi:10.1086/431524

For peer review only

For peer review only - http://bmjopen.bmj.com/site/about/guidelines.xhtml

Figure 1. Predicted annual number of IPD cases by VT and NVT and epidemiological year (July to June) from 2018/19 to 2030/31 with a 40% reduction in PCV13 coverage during the two lockdown periods starting 23 March 2020 for two months and 5 November 2020 for six months obtained from the long-term simulation model: A. Under 5 year olds and B. 65+ year olds without any reduction in contact rates: C. Under 5 year olds and D. 65+ year olds with 10% reduction in contact rates: E. Under 5 year olds and F. 65+ year olds with 40% reduction in contact rates: G. Under 5 year olds and H. 65+ year olds with 74% reduction in contact rates. Results show median and minimum and maximum of the range of model outputs generated by 500 model parameter sets [10]. (IPD, Invasive pneumococcal disease; VT, Vaccine types; NVT, Non-vaccine types)

Figure 2. Predicted median difference between the scenario without any change in PCV13 coverage and social mixing, and another scenario without any reduction in social mixing and no PCV13 vaccination during the two lockdowns in 2020/21 in the annual number of IPD cases, by serotype grouping (VT, NVT and overall) in all ages in England and Wales. (IPD, Invasive pneumococcal disease; VT, Vaccine types; NVT, Non-vaccine types)

For peer review only

For peer review only - http://bmjopen.bmj.com/site/about/guidelines.xhtml

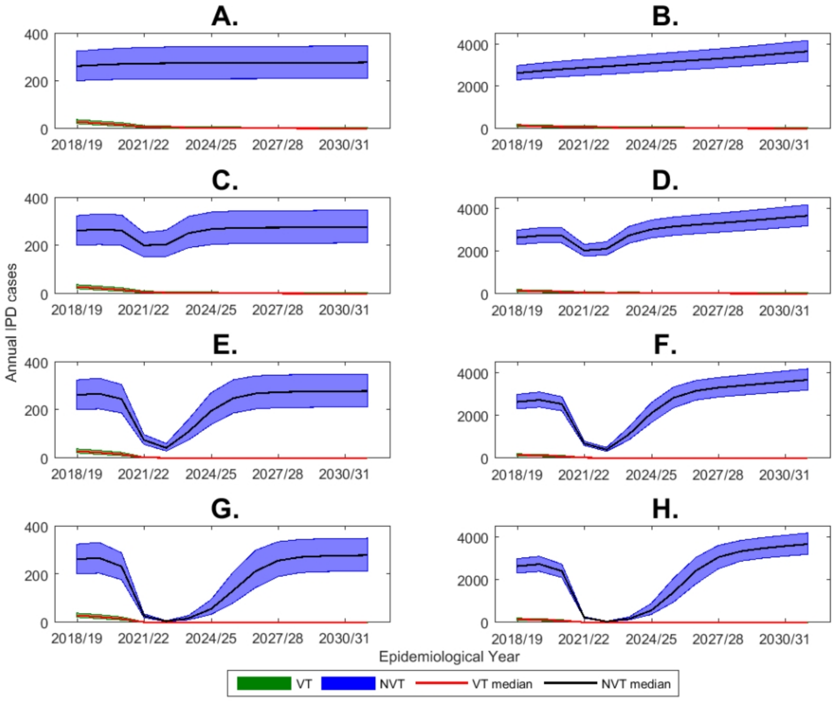

Figure 1. Predicted annual number of IPD cases by VT and NVT and epidemiological year (July to June) from 2018/19 to 2030/31 with a 40% reduction in PCV13 coverage during the two lockdown periods starting 23 March 2020 for two months and 5 November 2020 for six months obtained from the long-term simulation model: A. Under 5 year olds and B. 65+ year olds without any reduction in contact rates: C. Under 5 year olds and D. 65+ year olds with 10% reduction in contact rates: E. Under 5 year olds and F. 65+ year olds with 40% reduction in contact rates: G. Under 5 year olds and H. 65+ year olds with 74% reduction in contact rates. Results show median and minimum and maximum of the range of model outputs generated by 500 model parameter sets [10]. (IPD, Invasive pneumococcal disease; VT, Vaccine types; NVT, Non-vaccine types)

101x76mm (300 x 300 DPI)

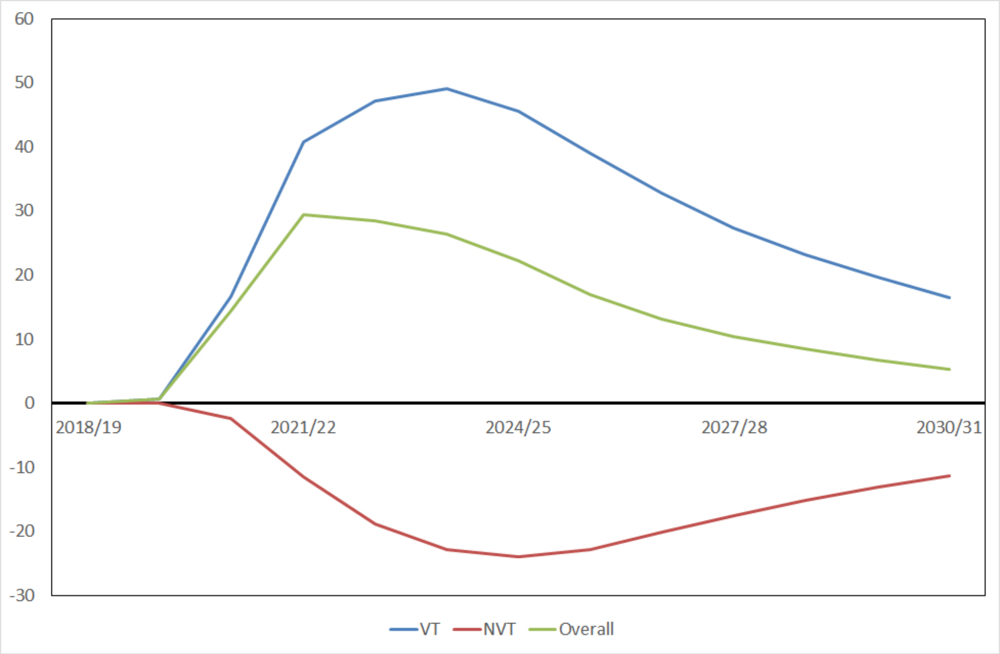

Figure 2. Predicted median difference between the scenario without any change in PCV13 coverage and social mixing, and another scenario without any reduction in social mixing and no PCV13 vaccination during the two lockdowns in 2020/21 in the annual number of IPD cases, by serotype grouping (VT, NVT and overall) in all ages in England and Wales. (IPD, Invasive pneumococcal disease; VT, Vaccine types; NVT, Non-vaccine types)

82x54mm (300 x 300 DPI)

Supplementary Appendix

Table S1. Cumulative difference in cases of invasive pneumococcal disease over 5 epidemiological years (July to June) starting from 2019/2020 by age group with different levels of reduction in PCV13 coverage and in contact rates during two lockdown periods starting 23 March for two months and 5 November 2020 for six months. Results show median and minimum and maximum of the range of model outputs generated by 500 model parameter sets [1]

Contact rate reduction	Vaccine coverage reduction	<2 yrs	2-4 yrs	5-14 yrs	15-44 yrs	45-64 yrs	65+ yrs	Total
0%	40%	3 (2, 5)	0 (0, 1)	0 (0, 1)	4 (2, 7)	4 (2, 7)	6 (2, 13)	18 (8, 33)
	100%	12 (6, 20)	2 (1, 3)	1 (1, 2)	16 (9, 26)	17 (8, 29)	25 (9, 50)	73 (33, 130)
10%	0%	-130 (-161, -97)	-47 (-57, -33)	-79 (-102, -74)	-617 (-697, -577)	-1021 (-1243, -890)	-2136 (-2484, -1837)	-4032 (-4489, -3606)
	40%	-128 (-159, -95)	-47 (-57, -33)	-79 (-102, -74)	-615 (-693, -575)	-1018 (-1240, -887)	-2130 (-2480, -1834)	-4027 (-4482, -3604)
	100%	-122 (-154, -88)	-46 (-56, -32)	-78 (-101, -73)	-607 (-685, -568)	-1009 (-1230, -880)	-2118 (-2459, -1825)	-3991 (-4432, -3566)
40%	0%	-471 (-580, -348)	-169 (-208, -118)	-275 (-349, -256)	-2062 (-2292, -1915)	-3368 (-4062, -2931)	-7107 (-8135, -6201)	-13494 (-14686, -12221)
	40%	-471 (-578, -347)	-169 (-208, -118)	-275 (-349, -256)	-2061 (-2291, -1913)	-3367 (-4062, -2931)	-7107 (-8135, -6201)	-13494 (-14676, -12211)
	100%	-468 (-576, -345)	-169 (-207, -117)	-275 (-349, -256)	-2059 (-2288, -1912)	-3366 (-4062, -2930)	-7107 (-8125, -6201)	-13484 (-14676, -12211)
74%	0%	-618 (-768, -447)	-221 (-280, -152)	-356 (-453, -329)	-2626 (-2890, -2393)	-4279 (-5183, -3690)	-9033 (-10248, -7910)	-17209 (-18511, -15541)
	40%	-617 (-768, -446)	-221 (-280, -152)	-356 (-453, -329)	-2626 (-2890, -2393)	-4279 (-5183, -3690)	-9033 (-10248, -7910)	-17209 (-18511, -15540)
	100%	-617 (-767, -446)	-221 (-280, -152)	-356 (-453, -329)	-2626 (-2890, -2392)	-4279 (-5183, -3690)	-9033 (-10248, -7910)	-17189 (-18511, -15540)

For peer review only

Table S2. Simulation results for the number of overall IPD cases between February and June 2020 and proportional reduction compared to the model predictions for the same period in 2019 (2,350 (UI 2,129, 2,531) IPD cases) in England and Wales according to different reduction levels in contact rates and vaccine coverage during two lockdowns due to COVID-19 in the UK. Results show median and minimum and maximum of the range of model outputs generated by 500 model parameter sets [1]

Contact rate reduction	Vaccine coverage reduction	IPD cases February to June 2020	Proportional reduction compared with February to June 2019
0%	40%	2,374(2,146, 2,554)	-0.9%(-1.3%, -0.6%)
	100%	2,375(2,148, 2,555)	-1.0%(-1.3%, -0.7%)
10%	0%	1,953(1,766, 2,101)	16.9%(16.5%, 17.3%)
	40%	1,954(1,766, 2,102)	16.9%(16.5%, 17.3%)
	100%	1,955(1,767, 2,103)	16.9%(16.5%, 17.2%)
40%	0%	1,125(1,017, 1,211)	52.2%(51.8%, 52.4%)
	40%	1,125(1,017, 1,211)	52.1%(51.8%, 52.4%)
	100%	1,126(1,018, 1,212)	52.1%(51.8%, 52.4%)
74%	0%	677(612, 728)	71.2%(71.1%, 71.3%)
	40%	677(612, 728)	71.2%(71.1%, 71.3%)
	100%	677(612, 729)	71.2%(71.1%, 71.3%)

For peer review only

For peer review only - http://bmjopen.bmj.com/site/about/guidelines.xhtml

Model description

Choi et al. [1] developed an age-structured, deterministic model structure to describe the pneumococcal transmission dynamics in England and Wales in order to estimate the potential impact of revising the 13-valent pneumococcal conjugate vaccine schedule from 2+1 to 1+1 in England and Wales. The data sources, model description, parameter estimation and fitting procedures described in this supplementary Appendix are a summary of those from Choi et al. [1]

Serotype groupings

In the model serotypes are grouped according to whether they are covered by the seven-valent pneumococcal conjugate vaccine (PCV7, VT1), or are one of the additional serotypes covered by PCV13 (VT2) or are non-vaccine serotypes (NVT). As in Choi et al [1] serotype 1 was excluded from the VT2 group as its incidence fell progressively after introduction of PCV7 in 2006 even though serotype 1 was not covered by PCV7, indicating secular changes in the behaviour of this serotype that are unrelated to vaccination. Further reductions in serotype 1 occurred after PCV13 introduction with continuing very low incidence [1] ; its exclusion from the model will therefore have a negligible impact on future IPD incidence. Serotype 3, which is also covered by the additional serotypes in PCV13, has increased progressively since 2013/14 behaving like a non-PCV13 serotype that exhibits serotype replacement. It was therefore included in the model in the NVT group [1]. For the current analysis, vaccine serotype outputs are shown combined as a single VT group though in the fitting stage VT1 and VT2 are separated to reflect the different invasiveness potential and propensity to induce serotype replacement of the VT1 and VT2 serotypes, and the differences between PCV7 and PCV13 vaccines in protecting respectively against carriage acquisition of VT1 and VT2 serotypes.

Model data

For peer review only

For peer review only - http://bmjopen.bmj.com/site/about/guidelines.xhtml

The datasets used for this modelling study include carriage prevalence data during the pre-PCV era, annual population changes by age, annual IPD cases by age groups and serogroups in pre- and post PCV introduction up to 2015/16 in England and Wales, PCV coverage data, and contact patterns in the population.

1. Carriage prevalence and IPD incidence

The pre-PCV7 carriage prevalence in seven age groups (under 1, 1-2, 3-4, 5-9, 10-19, 20-39 and 40+ years) and the three serotype groupings VT1, VT2 and NVT was obtained from a longitudinal family study [2] in which 3,869 nasopharyngeal swabs were taken from 489 individuals who were swabbed monthly over ten months between 2001 and 2002 in England.

The national enhanced surveillance dataset of serotyped IPD cases collated by Public Health England for epidemiological years (July to June) between 2000/01 and 2005/6 was used for the pre-PCV7 baseline incidence and the epidemiological years from 2005/6 to 2015/16 as the post-PCV period for the purposes of model fitting. The carriage prevalence and IPD datasets are available at https://doi.org/10.1371/journal.pmed.1002845.s001.

2. Population structure and contact patterns

The annual population size by age in each year from the pre-PCV7 to the post-PCV13 period was obtained from census data and the estimated demographic changes out to 2030 from the Office of National Statistics [3]. The contact pattern within and between age groups was derived from the POLYMOD survey conducted in the UK in 2006 [4] supplemented by an additional contact survey among infants under one year [5].

3. PCV coverage data

Vaccine coverage by dose, monthly birth cohort and calendar month in the PCV7 catch-up and the routine 2+1 PCV7 programme up to 2008 was obtained from the General Practice Research database

For peer review only - http://bmjopen.bmj.com/site/about/guidelines.xhtml

For peer review only

as previously described [6]. Thereafter, coverage for the second priming dose and the booster dose was obtained from annual national coverage data [7].

Model structure

1. Model population

The population in the model is divided into 100 annual age cohorts (0, 1, 2, 3, … , 99). Each annual age-cohort is divided into 48 equal-sized age-cohorts (in total 4,800 age-cohorts in the total population in the model).

2. Transition between model compartments

In the absence of vaccination individuals are born susceptible (S) to pneumococcal carriage and become infected (I) with a VT1, VT2 or NVT serotype as determined by the serogroup-specific force of infection. An episode of carriage does not result in protection against subsequent carriage of any serotype (i.e. SIS model structure). Invasive disease is assumed to occur at the time of carriage acquisition whereas transmission can occur at any time during the carriage episode. Individuals clear their infection with age-dependent clearance rates as estimated previously [8] and become susceptible again. Individuals already carrying a serotype from one of the three groupings will have some degree of protection against infections from another serotype grouping according to the level of competition between the three serotype groupings. These competition parameters determine the extent of serotype replacement in carriage (and therefore in IPD) when vaccine serotypes decline post-PCV introduction. The flowchart for the model structure is available at https://doi.org/10.1371/journal.pmed.1002845.g002.

3. Vaccine efficacy parameters

Within the vaccine protected group, two doses in the first year of life or one dose after 12 months of age are assumed to confer the maximum degree of protection that can be obtained from PCV7 or

For peer review only - http://bmjopen.bmj.com/site/about/guidelines.xhtml

PCV13 against carriage acquisition of the respective serotype grouping (termed full protection). A single dose in the first year of life is assumed to provide half the maximum protection (termed partial protection). Protection wanes exponentially with fully protected individuals moving back to the partially protected group and then back to the fully susceptible group. For the base case the average duration of protection for both full and partial protection is set at 5 years.

Vaccine efficacy against IPD given carriage of a vaccine serotype for fully protected individuals is assumed to be 100% at the time of vaccination and to wane with the same average duration as for the full and partial protection respectively against carriage. The flowchart showing the transitions between the unvaccinated, partially protected and fully protected compartments as a result of vaccination and subsequent waning is available at https://doi.org/10.1371/journal.pmed.1002845.g003.

The propensity to develop invasive disease upon carriage acquisition within each age group will be determined by the overall case-carrier ratio (CCR) of serotypes comprising the VT1, VT2 and NVT groupings (https://doi.org/10.1371/journal.pmed.1002845.g006) and is derived from the ratio of IPD cases: incident carriage infections within each serotype grouping in each age group.

Model parameter estimation

The model fitting process consists of a static pre-vaccination equilibrium component and post-vaccination dynamic component. By fitting the model to the given datasets, we estimate 26 model parameters: competition parameters for six age groups and three serotype groupings, the two vaccine efficacies against carriage acquisition of VT1 and VT2 serotypes and an additional parameter that allows for an increase in the case carrier ratio of the serotypes in the NVT group after 2013/14 when the rate of serotype replacement suddenly increased [9]. This last parameter is allowed to vary between each of the six age groups generating in total 26 parameters. A summary

For peer review only - http://bmjopen.bmj.com/site/about/guidelines.xhtml

table showing the derivations of the model parameters is available at https://journals.plos.org/plosmedicine/article/figure?id=10.1371/journal.pmed.1002845.t001.

As described in Choi et al [1] the force of infection and clearance parameters that determine the rate of acquisition and termination of a new carriage episode for the VT1, VT2 and NVT groups were estimated by fitting a static model to the pre-vaccination carriage data. The competition parameters that determine the extent to which carriage of serotypes in one group protects against acquisition of serotypes in another group were estimated by fitting a post-vaccination model to the changes in serotype grouping after the introduction of PCV7 and PCV13 from 2005/6 to 2015/16.

The uncertainty intervals were generated from 500 randomly selected sets of model parameters that generated outputs within ±0.3 of the set that gave the maximum likelihood value obtained using the Nelder-Mead Downhill Simplex method [10] when compared with the post-PCV IPD data. Results are shown as the median value of the accepted 500 parameters sets and the minimum to maximum range is shown as the uncertainty interval (UI) at https://journals.plos.org/plosmedicine/article/figure?id=10.1371/journal.pmed.1002845.t002. VEcVT1 (Vaccine efficacy against acquiring VT1 carriage) and VEcVT2 (Vaccine efficacy against acquiring VT2 carriage) were estimated at 0.55 (0.53, 0.57) and 0.30 (0.26, 0.36) respectively (minimum and maximum).

Long-term simulations

Using the 500 parameter sets obtained for the uncertainty boundary the model simulated various scenarios to investigate the potential impact of two lockdowns occurred during the COVID-19 pandemic until 2030/31, the furthest year for which there are population age-structure predictions.

References

1 Choi YH, Andrews N, Miller E. Estimated impact of revising the 13-valent pneumococcal conjugate vaccine schedule from 2+1 to 1+1 in England and Wales: A modelling study. *PLoS*

For peer review only - http://bmjopen.bmj.com/site/about/guidelines.xhtml

Med 2019;**16**. doi:10.1371/journal.pmed.1002845

2 Hussain M, Melegaro A, Pebody RG, *et al.* A longitudinal household study of Streptococcus pneumoniae nasopharyngeal carriage in a UK setting. *Epidemiol Infect* 2005;**133**:891–8.

3 Office for National Statistics. England Wales population size. https://www.ons.gov.uk/

4 Mossong JJ, Hens N, Jit M, *et al.* Social contacts and mixing patterns relevant to the spread of infectious diseases. *PLoS Med* 2008;**5**:e74.

5 van Hoek AJ, Andrews N, Campbell H, *et al.* The social life of infants in the context of infectious disease transmission; social contacts and mixing patterns of the very young. *PLoS One* 2013;**8**:e76180.http://www.pubmedcentral.nih.gov/articlerender.fcgi?artid=3797797&tool=pmcentrez&rendertype=abstract (accessed 30 Sep 2014).

6 Choi YH, Jit M, Gay N, *et al.* 7-Valent Pneumococcal Conjugate Vaccination in England and Wales: Is It Still Beneficial Despite High Levels of Serotype Replacement? *PLoS One* 2011;**6**:e26190.

7 Public Health England. Vaccine uptake guidance and the latest coverage data. 2018.https://www.gov.uk/government/collections/vaccine-uptake

8 Melegaro A, Gay NJ, Medley GF, *et al.* Estimating the transmission parameters of pneumococcal carriage in households. *Epidemiol Infect* 2004;**132**:433–41.

9 Ladhani SN, Collins S, Djennad A, *et al.* Rapid increase in non-vaccine serotypes causing invasive pneumococcal disease in England and Wales, 2000-17: a prospective national observational cohort study. *Lancet Infect Dis* 2018;**18**:441–51.

10 Nelder JA, Mead R. A Simplex Method for Function Minimization. *Comput J* 1965;**7**:308–13. doi:10.1093/comjnl/7.4.308

For peer review only
